# Near-Infrared Spectroscopy Used During Cardiopulmonary Resuscitation: Instrumentation, Signal Metrics, Clinical Context, and Feasibility: A Scoping Review

**DOI:** 10.3390/s26072136

**Published:** 2026-03-30

**Authors:** Zahra Askari, Mehdi Nourizadeh, Jacob Hutton, Sumaiya Hossain, Calvin Kuo, Jim Christenson, Brian Grunau, Babak Shadgan

**Affiliations:** 1 School of Biomedical Engineering, University of British Columbia, Vancouver, BC V6T 1Z4, Canada; askariz@student.ubc.ca (Z.A.); hsumaiya@student.ubc.ca (S.H.); calvin.kuo@ubc.ca (C.K.); 2 International Collaboration on Repair Discoveries, Vancouver, BC V5Z 1M9, Canada; mehdi22@mail.ubc.ca; 3 Department of Orthopaedics, University of British Columbia, Vancouver, BC V6T 1Z4, Canada; 4 Department of Emergency Medicine, University of British Columbia, Vancouver, BC V6T 1Z4, Canada; jacob.hutton@ubc.ca (J.H.); jim.christenson@ubc.ca (J.C.); brian.grunau@ubc.ca (B.G.)

**Keywords:** near-infrared spectroscopy, cardiopulmonary resuscitation, cardiac arrest, cerebral oximetry, optical sensing, monitoring

## Abstract

Conventional cardiopulmonary resuscitation (CPR) is guided primarily by process metrics that do not directly quantify cerebral hemodynamics or perfusion. Near-infrared spectroscopy (NIRS) provides continuous, non-invasive monitoring of regional tissue oxygenation and has emerged as a candidate modality for physiologic feedback during low-flow states. However, CPR applications vary across devices and signal processing. This scoping review maps how NIRS has been implemented during conventional CPR in humans and porcine models, with emphasis on instrumentation characteristics, signal processing, acquisition bandwidth, artifact handling, physiologic associations, and feasibility constraints. From 1048 records, 39 studies met the inclusion criteria. Most used forehead-based cerebral rSO_2_ monitoring (30/39). Rising cerebral oxygenation trajectories were consistently associated with return of spontaneous circulation (ROSC). In contrast, persistently low or non-increasing patterns were associated with non-ROSC, and absolute thresholds varied substantially across devices and studies. A minority of investigations derived compression-rate or waveform features from hemoglobin signals. Feasibility findings emphasized rapid probe placement without interrupting compressions but highlighted motion artifact, workflow constraints, and incomplete acquisition reporting. Overall, during conventional CPR, NIRS primarily serves as a dynamic monitor of oxygenation trends rather than a validated prognostic tool. Emerging waveform-based and hemodynamic analyses suggest the potential to evaluate CPR efficiency using perfusion-responsive optical features.

## 1. Introduction

Sudden cardiac arrest continues to result in low survival and substantial neurological disability despite advances in both basic and advanced life support. Patient outcomes remain tightly coupled to the quality of cardiopulmonary resuscitation (CPR) delivered during resuscitation. Process metrics, including compression depth, rate, fraction, limited peri-shock pauses, full recoil, and avoidance of hyperventilation, are associated with return of spontaneous circulation (ROSC) and survival to discharge; yet many individuals treated with guideline-compliant CPR do not survive. A major limitation of process metrics is that they do not directly reflect perfusion at the organ most vulnerable to ischemic injury [1,2,3,4]. This gap has renewed interest in real-time physiologic monitoring that focuses on cerebral oxygen delivery during resuscitation [5]. Continuous, brain-directed assessment is appealing because neurological outcome depends on oxygenation during low-flow states, whereas commonly used surrogates, including clinical examination, intermittent rhythm checks, end-tidal carbon dioxide, and invasive arterial pressure when available, do not measure cerebral hemodynamics and are susceptible to confounding by ventilation strategy, airway condition, and pauses in compressions for rhythm analysis [6,7].

Near-infrared spectroscopy (NIRS) is a non-invasive optical technique for monitoring regional tissue oxygenation and hemodynamics by quantifying the differential absorption of near-infrared light by oxy- and deoxyhemoglobin within the microvasculature. Light in the near-infrared spectrum penetrates biological tissue and is absorbed by hemoglobin chromophores, allowing concentration changes to be extracted through software-based algorithms grounded in modifications of the Beer–Lambert law, thereby yielding real-time measures of oxygenation and hemodynamics. In clinical settings, NIRS is most commonly applied to the forehead for continuous monitoring of cerebral oxygenation by sampling the superficial frontal lobe microvasculature [5,8]. Because optical signals can be acquired during low-flow or nonpulsatile states, when conventional monitors lose fidelity, cerebral NIRS offers an intuitively compatible physiologic modality for cardiac arrest and conventional CPR [6]. Commercial NIRS systems differ in wavelength sets, algorithmic processing, calibration approaches, and strategies to mitigate extracranial contamination, introducing between-device variation in absolute values and in proposed thresholds [7].

Over the past two decades, clinical and experimental investigations have explored several roles for NIRS during CPR. In real-time monitoring, compression-synchronized hemoglobin absorption waveforms and tissue oxygenation indices have tracked interruptions and variations in compressions, aligning optical metrics with the mechanics of resuscitation [5]. Translational porcine arrest models have demonstrated correlations between cerebral regional oxygen saturation (rSO_2_) and carotid blood flow or microcirculatory indices during compressions, supporting the physiological plausibility of NIRS as a marker of CPR quality [9]. Observational in-hospital cohorts have reported higher mean rSO_2_ values and rising trajectories in patients who achieve return of spontaneous circulation (ROSC) compared with non-ROSC cases [10], and a systematic review with meta-analysis has described similar directional associations while emphasizing heterogeneity in devices, timing, and thresholds [11]. In out-of-hospital cardiac arrest, rSO_2_ measured on hospital arrival has been investigated as an early marker of neurological outcome; however, although higher arrival values have been reported in patients with favourable outcomes, heterogeneity in the evidence limits its reliability as a stand-alone prognostic measure at presentation [11]. Emergency department have demonstrated feasibility during ongoing compressions and have noted that rSO_2_ frequently parallels short-term outcomes, including ROSC and survival to 6–24 h, although device-related and practical constraints remain [12,13]. Across animal and peri-arrest human observations, rSO_2_ generally decreases at arrest and increases with effective compressions and return of circulation, in alignment with mechanistic expectations for brain-directed resuscitation [9,14].

Despite these findings, the evidence base for cerebral and somatic NIRS during CPR remains fragmented across device types, wavelength sets, sampling rates, anatomic sites, and physiologic interpretations. Existing studies vary widely in methodology, sample size, and clinical context, obscuring how NIRS has been used in practice, which signal families may be most informative, and whether consistent physiologic patterns emerge across settings. Although research interest has grown, no synthesis has mapped how NIRS has been applied during CPR across devices, settings, signal families, acquisition strategies, and physiologic applications. A scoping review is therefore needed to systematically characterize the breadth of existing evidence and identify conceptual and methodological gaps that require clarification before future trials can evaluate NIRS-guided resuscitation [15].

The objective of this review is to examine how NIRS has been used during active, conventional CPR across preclinical, pediatric, and adult studies in prehospital, emergency department, and in-hospital settings, with emphasis on instrumentation characteristics, signal types, physiologic associations, clinical applications, and feasibility constraints. Accordingly, this scoping review addressed how NIRS has been used as a physiologic monitor during conventional CPR in humans and porcine models, focusing on three domains:

1. Which NIRS devices, acquisition parameters, signal types, and signal-processing approaches have been reported during conventional CPR, and how completely are these parameters captured across the literature?
2. What NIRS-derived metrics, thresholds, and quantitative performance measures have been associated with ROSC, CPR quality, or hemodynamic surrogates; what functional roles were described for NIRS (predictive, confirmatory, or CPR-quality feedback), and how did these associations vary across devices, timing windows, and resuscitation contexts?
3. How do reported clinical applications, outcomes, and feasibility constraints vary by arrest setting, patient population, and study model, and what evidence gaps and limitations recur across the literature?

## 2. Materials and Methods

This scoping review was conducted in accordance with the PRISMA Extension for Scoping Reviews (PRISMA-ScR) and guided by the Joanna Briggs Institute (JBI) methodology, using the Population–Concept–Context (PCC) framework to define eligibility criteria and organize data charting [16,17].

### 2.1. Eligibility Criteria

Population—We included humans of any age, except newborns, receiving conventional cardiopulmonary resuscitation with manual chest compressions. Eligible preclinical studies included porcine models of cardiac arrest treated with conventional CPR. Non-porcine species were excluded to maintain comparability of cranial and soft tissue optical paths during compressions.

Concept—Eligible studies used NIRS systems during the CPR epoch to monitor cerebral or somatic oxygenation, inform resuscitation decisions, estimate the likelihood of ROSC or short-term survival, and describe physiologic responses to compressions. Any device and output qualified, including regional cerebral oxygen saturation (rSO_2_), tissue oxygen saturation (StO_2_), tissue oxygenation index (TOI), total hemoglobin concentration, and absorption-based metrics such as changes in oxygenated hemoglobin (O_2_Hb), deoxygenated hemoglobin (HHb), and total hemoglobin (THb).

Context—Settings included prehospital care, emergency departments, intensive care units, operating rooms, and animal laboratories. Eligibility was limited to the active CPR interval, from the start of compressions until return of spontaneous circulation or termination of efforts. Post-ROSC monitoring was out of scope. We excluded extracorporeal CPR due to its different circulatory physiology. Eligible designs included prospective and retrospective cohorts, interventional studies, feasibility or technical reports, case series with two or more cases, and experimental porcine studies. Reviews were used for background, not charted. We included English language peer-reviewed full texts. Case reports, conference abstracts, and proceedings were excluded. When reports covered CPR and post-ROSC periods, we extracted CPR-specific data and, in several cases, near-ROSC or post-ROSC time windows.

### 2.2. Information Sources and Search Strategy

We searched Embase, MEDLINE, PubMed, and CINAHL using a combination of controlled vocabulary (e.g., Near-Infrared Spectroscopy, Cardiac Arrest, Cardiopulmonary Resuscitation) and free-text keywords. Keyword strings included variations of “near-infrared spectroscopy,” “NIRS,” “cerebral oximetry,” “regional cerebral oxygen saturation,” “tissue oxygenation index,” “oxygenated hemoglobin,” “deoxygenated hemoglobin,” “total hemoglobin,” “O_2_Hb,” “HHb,” “rSO_2_”, “TOI” combined with CPR-related terms (“cardiac arrest,” “cardiopulmonary resuscitation,” “CPR,” “out-of-hospital cardiac arrest,” “in-hospital cardiac arrest,” “OHCA,” “IHCA”). Each strategy was adapted to the database using appropriate subject headings and field tags. All records were exported to a unified library, deduplicated, and prepared for screening.

### 2.3. Selection Process

**Figure 1:**
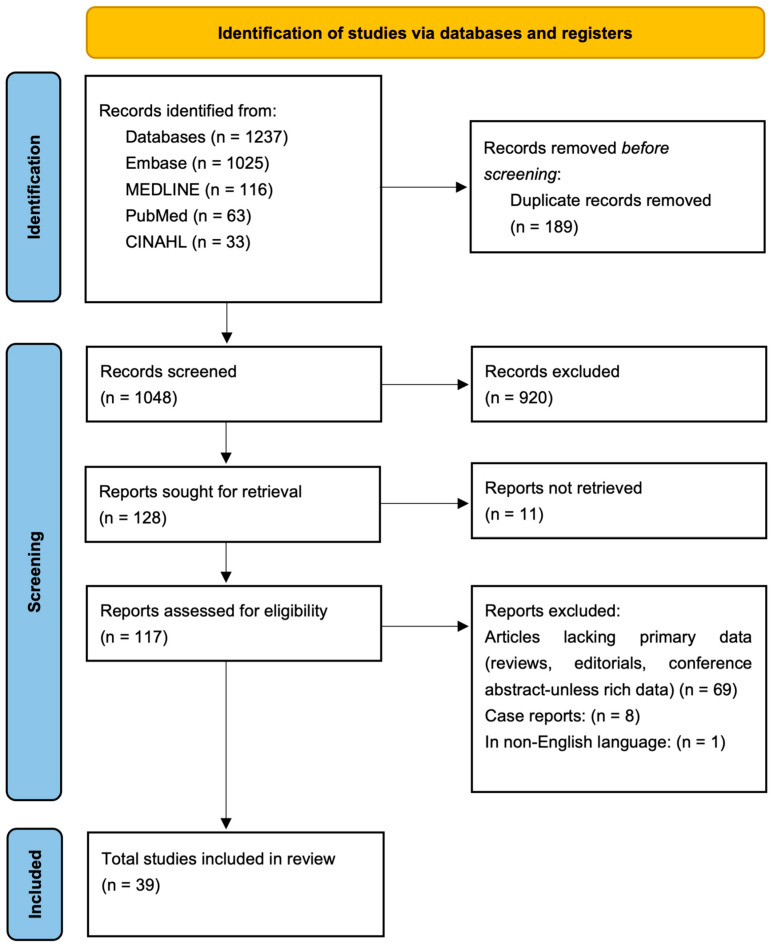
PRISMA 2020 flow diagram showing included studies at each stage of the inclusion/exclusion process. Databases include Embase, MEDLINE, PubMed, and CINAHL. n: Number of papers reviewed by the authors at each step.

Screening occurred in two stages: title and abstract review, then full-text assessment, using the prespecified Population–Concept–Context (PCC) criteria. We documented reasons for exclusion in the full text. Two reviewers independently screened all records and resolved disagreements by consensus. Screening and decision tracking were managed in Rayyan [18], and the study selection process was summarized in a PRISMA-ScR flow diagram (Figure 1) constructed in Microsoft Word (version 16.105.2).

### 2.4. Data Extraction

Two reviewers independently extracted and charted data from each included study using a standardized extraction form, with disagreements resolved through discussion and iterative calibration to ensure consistent interpretation. Extracted variables were organized into four study-level categories. (1) Core study characteristics, including the study identifier (first author/year), study design, optical application, setting, episode type, witnessed status, bystander CPR, initial rhythm, population, age (years), proportion of male participants, and sample size. (2) NIRS instrumentation and acquisition parameters, including the device brand/model, wavelengths (nm) when reported, number of sensors, sampling rate (Hz), whether the system was wired or wireless, and the sensor site. (3) Processing and synchronization details and the contextual framing of NIRS measurement during CPR, including the metric family (regional cerebral oxygen saturation, tissue oxygenation index, and related hemoglobin-derived metrics), timing window, compression context and compression ratio (when described), the method used to align NIRS signals with CPR epochs, artifact handling approaches, reported data loss (%), software or toolbox used, and the exposure of interest or NIRS feature under evaluation. (4) Each study’s purpose and interpretation, including the stated aim, primary and main findings, feasibility notes relevant to implementation during resuscitation, and author-reported limitations.

### 2.5. Synthesis

We synthesized results descriptively, summarizing NIRS applications during CPR, study settings, populations, instrumentation features, and CPR-epoch findings such as absolute values, trajectories, thresholds, and outcome associations. We did not perform meta-analysis due to study heterogeneity. To strengthen evidence interpretation, we grouped feasibility notes and author-reported limitations into recurrent domains of systematic error, such as selection limitations, measurement limitations, performance or detection concerns, residual confounding, and limited external validity. This was done as a structured narrative appraisal, not a formal risk-of-bias scoring exercise. To focus on conventional CPR physiology, we excluded ECPR studies based on established definitions for extracorporeal support during refractory cardiac arrest.

**Figure 2:**
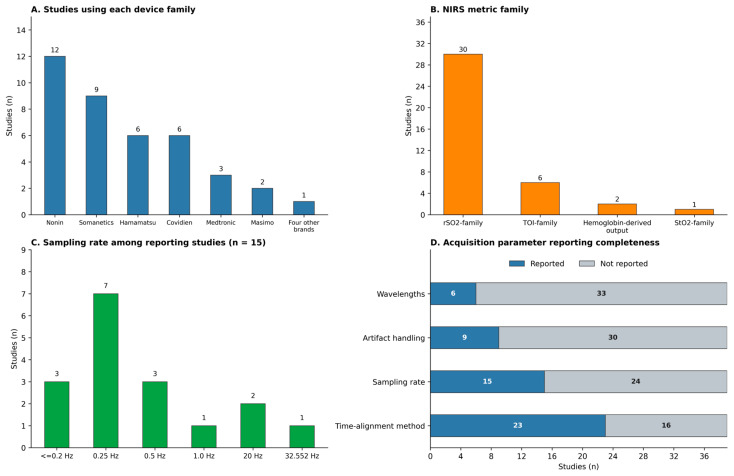
NIRS instrumentation characteristics and acquisition parameter reporting.

**Figure 3:**
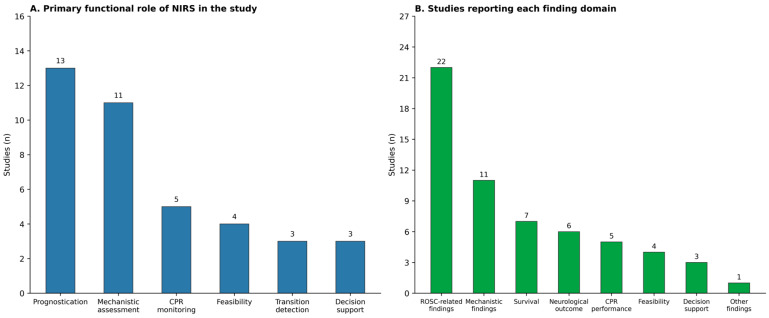
NIRS functional roles and outcome domains reported across studies.

**Figure 4:**
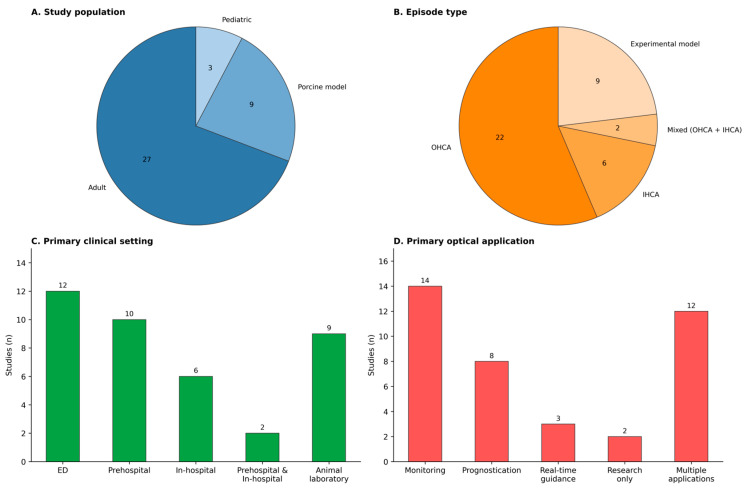
Study-level context of included studies (n = 39).

Data visualization and plotting for Figures 2–4 were conducted using Python (version 3.14.3) and Microsoft PowerPoint (version 16.107.1). Manuscript preparation and citation management were completed using Microsoft Word (version 16.105.2) and Zotero (version 7.0.32).

## 3. Results

### 3.1. Selection of Sources of Evidence

A total of 39 studies met eligibility criteria (Figure 1) and were charted in this scoping review (Table A1, Table A2, Table A3 and Table A4). The literature spanned 2006–2025, with 10 studies published from 2006 to 2014, 13 from 2015 to 2019, and 16 from 2020 to 2025, indicating an increase in publication volume in the most recent period.

### 3.2. Characteristics of Sources of Evidence

Across the 39 included studies, designs were predominantly non-randomized. Based on extracted study-design descriptions, 24 studies were classified as clinical observational, 3 as clinical interventional (randomized or non-randomized), 3 as case series, and 9 as experimental animal studies.

### 3.3. Instrumentation, Acquisition Parameters, Signal Types, and Reporting Completeness

Device use clustered around a small number of commercial NIRS systems. Across the 39 included studies, 17 used INVOS systems, reported as INVOS platforms: 9 from Somanetics (Troy, MI, USA), 3 from Medtronic (Boulder, CO, USA; Minneapolis, MN, USA), and 5 from Covidien (Mansfield, MA, USA; Boulder, CO, USA). Twelve studies used Nonin systems, such as the Equanox 7600, Equanox Advance monitor, and SenSmart Model X-100 Universal Oximetry System (Nonin Medical Inc., Plymouth, MN, USA). Six studies used Hamamatsu devices, including the NIRO-series in five studies and CCR-1 in one study (Hamamatsu Photonics, Hamamatsu City, Japan). Two studies used Masimo devices, namely the Root and O3™ Regional Oximeter and Masimo Open Connect (MOC-9) (Masimo Corporation, Irvine, CA, USA). FORE-SIGHT (CAS Medical Systems, Inc., Branford, CT, USA) and InSpectra (Hutchinson Technology Inc., Hutchinson, MN, USA) each appeared in one study. Three additional systems were each reported in a single study: the TOS-QQ® brain oximeter (TOSTEC Co., Ltd., Tokyo, Japan), a Covidien NIRS system (Covidien, The Netherlands), and NIRSIT ON (OBELAB Inc., Seoul, Republic of Korea). Because some studies employed more than one device family, the counts are not mutually exclusive (Table A2).

Explicit reporting of whether the NIRS sensor connection was wired or wireless was uncommon. Only one study [19] explicitly described the sensor-to-monitor interface as cabled. To characterize connectivity more systematically, reported device models were matched with publicly available manufacturer documentation. Using this approach, 36 of 39 studies were classified as using systems with cabled sensors, one study used a wireless system [20], and connectivity could not be confirmed for two studies [21,22] (Table A2).

Thirty one of the 39 studies positioned probes exclusively on the forehead or frontal region. Four studies combined forehead or frontal probes with a second, non-cranial site: the flank over the kidney [23], the thenar eminence of the hand [24], bilateral abdominal sites [25] or the thigh over a muscle [9]. The remaining four studies used alternative configurations: two described the placement only as “cerebral” [26,27]; one placed a probe on the parietal skull [28]; one monitored a non-cranial site alone (thenar eminence) without a cerebral probe [29] (Table A2).

Reported NIRS outputs were predominantly saturation-based measures, most often cerebral. Based on the extracted metric family, 30 of 39 studies reported an rSO_2_-family metric (including rSO_2_, cerebral oxygen saturation (ScO_2_), cerebral regional oxygen saturation (crSO_2_), and cerebral tissue oxygen saturation (SctO_2_) variants), six reported a TOI-family metric (tissue oxygenation index), one reported an StO_2_-family metric (thenar StO_2_), and two reported hemoglobin concentration or chromophore-change outputs without rSO_2_, TOI, or StO_2_ measures (Table A3).

Reporting of acquisition and processing characteristics required for cross-study comparability was frequently incomplete. Wavelengths were not reported in 33 studies, and the sampling rate in 24, whereas the number of sensors was not reported in only four. Short-separation channels or superficial regression methods were not reported in 35 studies. Time-alignment methods were not reported in 16 studies, artifact-handling methods in 30, and data loss in 25, limiting the interpretation of signal behaviour during motion-intensive compressions (Table A2 and Table A3). Temporal acquisition characteristics were reported in varying ways. Sampling rate was specified in 15 of 39 sources (38%). Of these, 11 used low-frequency continuous monitoring at 0.16–0.5 Hz, corresponding to approximately one data point every 2–6 s, including six studies sampling every four seconds (0.25 Hz). Two studies used higher sampling rates of 20 Hz or 32.552 Hz [20,30]. Two additional studies reported device- or processing-specific sampling characteristics, including one study that documented different sampling rates across two devices used in the same arrest cohort (0.25 Hz for INVOS and 1 Hz for Equanox) [31] and one study that described 0.5 Hz data that were recalibrated to 20 Hz for analysis [5]. In the remaining 24 studies (62%), sampling frequency was not reported (Table A2). Sampling frequency determines which temporal frequency components in a sampled waveform can be represented without distortion, as the Nyquist criterion requires sampling at least twice the highest frequency component of interest; sampling below that rate produces aliasing (frequency folding) [32]. When rSO_2_ (or a related NIRS index) is recorded at 0.16–0.5 Hz (Δt ≈ 2–6 s, where t_s = 1/f_s), the corresponding Nyquist frequency is only 0.08–0.25 Hz. Consequently, oscillations at chest-compression frequencies near the guideline compression rate of 100–120/min (≈1.7–2.0 Hz) [33] cannot be resolved without aliasing. Such recordings, therefore, primarily support interpretation of slower, trend-level changes in the mean value rather than compression-synchronous pulsatility.

The type of chest compressions used in the studies was inconsistently documented. Based on the extracted reports, 8 studies were classified as manual-only, 6 as mechanical-only, and 9 as manual–mechanical comparisons; 16 did not report the compression context. When methods were described, time alignment was more commonly reported than artifact handling: 23 studies reported a time-alignment method, 9 reported explicit artifact-handling methods, and 14 reported data loss (Table A3).

In summary, addressing our first research question reveals that the literature was dominated by a limited number of named NIRS platforms and by cerebral saturation-based outputs, but that cross-study interpretability remained constrained by incomplete reporting of acquisition, preprocessing, and synchronization details. These omissions are especially important when studies are compared on the basis of absolute values, thresholds, or device-specific behaviour rather than within-study trajectories. The distribution of device families, NIRS metric families, reported sampling frequencies, and acquisition-parameter reporting completeness is summarized in Figure 2.

### 3.4. Physiologic Associations, Thresholds, Quantitative Performance, and Functional Roles

NIRS served overlapping prognostic, confirmatory, and CPR-quality feedback roles across the included literature. Nineteen studies included a prognostication or outcome-association aim, 20 described NIRS as a physiologic monitoring tool during CPR, and six explicitly evaluated real-time guidance applications. These categories were not mutually exclusive. In addition, 10 studies described NIRS as research-only acquisition, and one further study, categorized as prognostication (research), similarly reflected a context in which NIRS data were not used to guide contemporaneous care [34]. When implementation details were reported, research-only acquisition was achieved by keeping displays out of view or instructing clinicians not to act on the values [29,35]. The dominant functional roles assigned to NIRS and the outcome domains reported across studies are summarized in Figure 3.

Across adult OHCA cohorts using rSO_2_-family metrics, the most consistent pattern was that ROSC cases showed higher or rising intra-arrest cerebral oxygenation, whereas non-ROSC cases showed persistently low or non-rising trajectories [13,14,36,37,38,39]. Nine adult OHCA studies plotted rSO_2_ values against resuscitation duration, and these reports, for example, described low initial values that increased among patients who achieved ROSC. A smaller number of studies examined whether NIRS could predict ROSC before circulation was restored. Prosen et al. described a rapid, sustained rise occurring minutes before ROSC with normalization after ROSC [36], whereas two other studies found that very early single values performed poorly as discriminators [34,37]. These findings support the use of NIRS more strongly as a dynamic marker of evolving circulation and ROSC-related transition than as a validated early predictive signal.

Quantitative thresholds and performance metrics were reported, but they varied by signal family, timing window, device context, and clinical endpoint. In witnessed emergency department cardiac arrests, rSO_2_ during CPR yielded an AUC of 0.74 for ROSC, with 100% sensitivity at a threshold of ≥24% and 100% specificity at ≥64% [12]. In adult emergency department arrest, a 30% rSO_2_ cut-off yielded sensitivity of 91.7%, specificity of 37.1%, positive predictive value of 50%, negative predictive value of 86.7%, and an AUC of 0.76 for ROSC [13]. In prehospital OHCA, a ΔTOI threshold of 5% yielded sensitivity 65.4% and specificity 89.3% for ROSC; ΔTOI values of ≥8% were observed only in ROSC, and ΔTOI values of ≤−2% only in non-ROSC [21]. In adult OHCA, highest-rSO_2_ AUCs ranged from 0.724 to 0.743 across the initial 5 min, initial 10 min, and overall windows, with corresponding cut-offs of 24%, 30%, and 26%; mean-rSO_2_ measures yielded lower AUCs of 0.677 to 0.724, and persistent overall rSO_2_ ≤ 18% was uniformly associated with non-ROSC [37].

Outcome-associated thresholds also varied across other populations and timing windows. For example, in adult OHCA assessed at emergency-department arrival during ongoing CPR, initial TOI improved ROSC discrimination (AUC 0.88) compared with lack of TOI data (AUC 0.79), and all patients with TOI ≥ 59% survived to hospital discharge whereas a TOI of ≤24% was associated with failure to achieve ROSC [40]. In pediatric IHCA, subgroup-specific median crSO_2_ cut-offs for ROSC ranged from 25.5% to 37.3% (AUC 0.69–0.85) across cyanotic heart disease strata [31]. For good neurological outcome after OHCA, an arrival rSO_2_ threshold of >42% yielded sensitivity of 0.79, specificity of 0.95, and an AUC of 0.90 for distinguishing outcomes [41]. Similarly, in adult IHCA, an rSO_2_ cut-off of 47.6% predicted ROSC (AUC 0.978, sensitivity 94%, specificity 92%), while a dynamic increase of 4 percentage points during a CPR loop predicted ROSC (sensitivity 80.4%, specificity 83.2%, AUC 0.875) [27]. Finally, in ED OHCA, a frontal rSO_2_ rise during CPR was associated with survival, discriminating between survivors and non-survivors (AUCs > 80%) [42].

When NIRS was used as a physiologic feedback tool rather than solely as an outcome-associated signal, associations with conventional hemodynamic surrogates were present but generally modest or context-dependent. For example, in an adult OHCA emergency-department cohort, log-transformed rSO_2_ and mean arterial pressure showed a mild but statistically significant association [43], and rSO_2_ correlated weakly with systolic and diastolic blood pressure, but not with PaO_2_, PaCO_2_, or ETCO_2_ [19]. Similarly, in a hypothermic porcine model, rSO_2_ tracked CPP and ScvO_2_ during stable compressions before adrenaline administration, but after adrenaline, CPP and PbtO_2_ increased while rSO_2_ remained unchanged [44]. In addition to these correlations, a smaller waveform-focused literature captured CPR-process information more directly. Koyama et al. reported compression-synchronous ΔcHb waveforms that tracked chest compressions in real time [5], while Sanz-Pescador et al. estimated chest compression rate from cerebral oximetry signals with a median absolute error of 0.62 compressions per minute [30]. Additionally, prehospital TOI change correlated with chest compression rate during ambulance transport [21]. Despite these findings, explicit workflow-guiding use remained uncommon. For instance, Tsukuda et al. evaluated TOI as a candidate aid for prehospital decision-making during resuscitation [21], Takegawa et al. incorporated rSO_2_ into a TripleCPR protocol without a clear ROSC benefit in the main IPTW analysis [22], and Kishihara et al. explicitly framed cerebral rSO_2_ as a quality indicator for chest compressions in the emergency department [43].

In addressing our second research question, the extracted data demonstrate that NIRS has been used both as an outcome-associated signal and as a physiologic feedback tool during CPR. The literature supports recurring ROSC-related trajectory patterns and several study-level thresholds with reported quantitative performance, but those findings varied substantially by output family, timing window, device context, and intended functional role. As a result, the evidence supports clinical relevance, but not a single generalized threshold or pooled sensitivity/specificity framework across the full corpus.

### 3.5. Clinical Context, Outcomes, and Feasibility Constraints

Adult populations accounted for most of the clinical evidence. Among human studies (n = 30), 27 enrolled adult participants, and three enrolled pediatric participants. Twenty-one human studies focused on OHCA, six on in-hospital cardiac arrest (IHCA), and three included both OHCA and IHCA episodes. Thirteen human studies were conducted in emergency department settings, 12 in prehospital settings (including prehospital-only and prehospital-to-emergency department (ED)/intensive care unit (ICU) pathways), and five in in-hospital or ICU settings (Table A1). Return of spontaneous circulation (ROSC) was the most frequently reported clinical endpoint, appearing in 26 of 39 studies. Survival outcomes were reported in seven studies and neurological outcomes in five; when reported, these endpoints typically appeared alongside ROSC rather than as stand-alone outcomes. In contrast, 12 of the 39 studies did not report ROSC, survival, or neurological outcomes, instead focusing on physiologic or methodological endpoints such as associations with arterial pressure, oxygenation, or ventilation parameters, hyperfibrinolysis, or compression-synchronous waveform features (Table A4). Collectively, this distribution of endpoints indicates that the evidence base is strongest for short-term ROSC-related monitoring in adult OHCA, with substantially less evidence for pediatric resuscitation exclusively in in-hospital settings and for longer-horizon outcomes. Study-level context, including study population, episode type, primary clinical setting, and primary optical application, is summarized in Figure 4.

Clinical application varied across populations and settings. Adult OHCA studies most often examined ROSC-associated trajectories, early-window monitoring, or arrival-based prognostication [14,34,36,37,40,41]. Pediatric evidence was limited. Two pediatric cohorts found that higher intra-arrest cerebral saturation was associated with ROSC [31,35], and one of them also linked higher crSO_2_ values and greater time above threshold to survival to discharge and favourable neurological outcome [31]. A separate pediatric OHCA report described cerebral oximetry with a blood volume index as feasible decision support when end-tidal CO_2_ changes were lost during resuscitation [45]. Adult in-hospital evidence was less extensive but included higher maximum rSO_2_ values among survivors in an ICU cohort [46] and quantitative ROSC and 30-day survival thresholds in an IHCA cohort [27]. In an adult OHCA emergency department cohort, rSO_2_ also showed a mild but statistically significant association with mean arterial pressure during CPR [43]. Animal studies contributed mechanistic or controlled intervention data rather than directly generalizable clinical thresholds, including site-dependent differences in the correlation between NIRS readings and brain tissue oxygen partial pressure [47], oxygen-fraction effects on rSO_2_ or PbtO_2_ [48,49], etiologic differences in TOI trajectories during CPR [50], and abrupt ROSC-related signal changes with moderate correlation to target cardiac output in a pediatric swine model [23].

Feasibility reporting was uneven, with six studies providing no specific feasibility note (Table A4). Where described, deployment ranged from 15 to 20 s during mechanical-device installation [12], less than 30 s in a prehospital feasibility cohort [38], and within one minute after hospital arrival [22]. An IHCA study reported median times of 5 (3–7) minutes for sensor placement and 15.5 (8.3–22.8) minutes for monitoring [51]. Practical constraints included the need for additional personnel [7], delayed or selective monitoring because of competing advanced life support priorities, limited space, attachment failure, insufficient personnel, probe shortages, or device and power constraints [21,27,36,41]. Research-only acquisition was also common. Several studies explicitly kept displays out of view or instructed teams not to act on the readings [29,34,35].

Finally, regarding our third research question, these findings reveal that NIRS has been applied across multiple populations and settings. However, the evidence remains unevenly distributed across clinical contexts and continues to be shaped by recurrent operational and methodological constraints.

### 3.6. Critical Appraisal of Sources of Evidence

A formal critical appraisal was not done. This aligns with the scoping review’s aim to map evidence, not to grade effect estimates. Nevertheless, extracted feasibility notes and study limitations enabled a structured narrative appraisal of recurring systematic-error domains. Selection-related limitations were common. NIRS was often initiated only after CPR began. Sometimes, it could be used only if arrests lasted long enough for probe placement [12,35,36]. Other cohorts had incomplete enrollment or selective monitoring due to personnel, equipment, or operational limits [39,41]. Measurement limitations were also frequent. These included device-specific floors, proprietary processing, possible extracerebral contamination, unilateral sensing, and limited artifact control [7,34,36,38,40,49]. These measurement challenges worsened with incomplete reporting. Of the 39 included studies, NIRS wavelengths were unreported in 33, sampling rates in 24, and artifact handling in 30. Feasibility notes were missing in 6 studies. Performance or detection concerns occurred when clinicians were not blinded to NIRS values or when NIRS influenced resuscitation decisions [22,39,41]. Other studies reduced this risk by masking the display or by instructing teams to ignore readings [14,29]. Residual confounding was hard to rule out when CPR quality, peri-resuscitation variables, or resuscitation timing were not fully captured [10,19,27,29,38,51]. External validity was further limited. Small, single-centre pilot cohorts and experimental porcine models using young, healthy animals in controlled conditions contributed to this [21,35,38,49]. These factors indicate that the mapped evidence is best interpreted as hypothesis-generating rather than confirmatory, particularly for prognostic thresholds or CPR-guiding claims.

### 3.7. Integrated Synthesis of Evidence

In summary, the mapped literature answers the three review questions in a consistent pattern. First, conventional CPR studies used a few NIRS platforms. These mainly relied on cerebral saturation-based outputs. However, acquisition and preprocessing details, such as wavelengths, channel configuration, sampling rate, superficial-signal handling, time alignment, and artifact processing, were often incompletely reported (Table A2 and Table A3). For the second question, rising cerebral oxygenation was repeatedly linked with ROSC-related trajectories [13,14,36,37,38,39]. Several studies provided thresholds or quantitative metrics for ROSC, survival, or neurological outcome [12,13,21,27,31,37,40,41,42]. These findings suggest clinical relevance but vary by output family, timing window, and endpoint. Therefore, a device-agnostic threshold or pooled sensitivity/specificity framework cannot be justified across the full corpus. Lastly, for the third domain, evidence was concentrated in adult OHCA and prehospital or emergency department settings. In contrast, pediatric cohorts, strictly in-hospital settings, and longer-term survival or neurological outcomes were rarely represented (Table A1 and Table A4).

Across these three domains, the literature most strongly supports two conclusions. First, NIRS can be deployed during ongoing CPR across multiple clinical settings. However, operational reliability and workflow burden remain inconsistent when described [7,12,21,22,27,36,38,41,51]. Second, NIRS is physiologically responsive during resuscitation. It repeatedly captures ROSC-related transition patterns [13,14,36,37,38,39]. Still, the generalizability of the interpretation of absolute values remains limited. Incomplete reporting, device comparability constraints, heterogeneous timing windows, and the predominance of observational or feasibility studies contribute to this (Table A2, Table A3 and Table A4). The main knowledge gaps, therefore, remain direct platform comparison, clearer separation of predictive versus confirmatory roles, more consistent quantitative performance reporting, and expansion into underrepresented contexts such as pediatric resuscitation, adult IHCA, and longer-horizon outcomes.

## 4. Discussion

Viewed through the three review domains, the mapped literature suggests that NIRS during conventional CPR is primarily an observational and physiologic monitoring field. It is not yet a mature interventional technology. The evidence is concentrated in adult OHCA and in prehospital or emergency department settings. The most consistent finding is an increase in cerebral oxygenation during ROSC. There is no reproducible device-agnostic threshold (Table A1, Table A2, Table A3 and Table A4; [13,14,36,37,38,39]).

### 4.1. Instrumentation, Device Heterogeneity, and Acquisition Reporting

With respect to our first research question, the literature appears more standardized at the level of named commercial platforms than at the level of analytically comparable measurement systems. Most studies used Nonin, Somanetics, Hamamatsu or Covidien devices and reported saturation-based cerebral outputs, but the corpus also included TOI, peripheral StO_2_, and hemoglobin-derived features, suggesting that apparently similar oxygenation values did not necessarily correspond to the same output family across studies (Table A2 and Table A3). Device heterogeneity complicates interpretation, as the heterogeneity captured in this review extends beyond brands to include underlying signal-processing approaches and output families [5,7,21,40]. Included studies explicitly cautioned that proprietary algorithms, calibration differences, and device-specific low-end behaviour may limit comparability across platforms [7,31,36,40]. In one pediatric multicenter analysis, calibration and threshold differences between INVOS and Equanox were identified as limiting the applicability of a single universal crSO_2_ target [31]. Values at the lower end of the measurement range may also not be strictly comparable: in one OHCA cohort, the INVOS display floor was 15%, with lower values rendered as zero [36], whereas a dual-device feasibility study reported extremely low EQUANOX values and raised the possibility of a technical artifact [7]. These findings suggest that within-platform trends may be more interpretable than device-agnostic absolute thresholds, particularly when cerebral oximetry is used for prognostication, or CPR-guidance claims [7,31,36,40]. This concern is reinforced by the review-level finding that wavelengths, channel configuration, sampling rate, superficial-signal handling, time alignment, and artifact-processing methods were frequently unreported (Table A2 and Table A3). When sampling frequency was reported, most studies used low-frequency acquisition at 0.16–0.5 Hz, whereas only a small subset used higher-frequency or waveform-oriented approaches [5,20,30]. One additional study reported different sampling rates across two devices used in the same arrest cohort [31]. This pattern is consistent with the predominance of trajectory-based analyses over CPR-specific waveform features in the current literature [5,30]. Signal interpretation is further conditioned by measurement site and tissue contributions, because several studies reported possible scalp or non-cerebral influences on the recorded signal [20,34,49], and one porcine study showed divergence between skin- and skull-based measurements, with only skull-based values correlating with brain tissue oxygen tension [47]. Taken together, Q1 suggests that the main methodological bottleneck is not the absence of available NIRS systems, but the limited comparability and incomplete reporting of how those systems acquired and processed the signals interpreted during CPR (Table A2 and Table A3; [7,31,36,40]).

### 4.2. Physiologic Interpretation, Quantitative Performance, and Clinical Role of NIRS

Across the current literature, three partially overlapping clinical roles for NIRS during conventional CPR can be distinguished: ROSC-related transition detection or confirmation, prognostication of survival or neurological outcome, and assessment of CPR quality or cerebral perfusion [14,21,27,31,34,36,37,38,39,40,41,43]. The strongest evidence supports the first of these roles [13,14,36,37,38,39]. Higher or rising intra-arrest cerebral oxygenation was repeatedly associated with ROSC-related patterns [13,14,36,37,38,39], whereas evidence that NIRS can reliably anticipate ROSC before circulation is restored remains more limited [14,34,36,37,40]. Prosen et al. described a sustained rise before ROSC [36], whereas other cohorts found that very early single values were poor discriminators or that more informative associations emerged only after aggregation across defined intra-arrest windows, at post-ROSC time points, or at hospital arrival [14,34,37,40,41]. Reports of favourable neurological outcome despite very low initial rSO_2_ values further argue against using early absolute measurements to declare futility [34].

Quantitative studies support clinical relevance, but there is no single pooled estimate of diagnostic performance. Reported thresholds and measurement metrics varied by output family, timing window, device context, and the outcome assessed. In adult OHCA, Jang et al. showed that discrimination varied depending on whether the highest or mean rSO_2_ values were analyzed and whether the early or overall windows were used [37]. Tsukuda et al. reported TOI-based thresholds rather than rSO_2_-based thresholds [21,40], Raymond et al. reported pediatric crSO_2_ associations using prespecified thresholds [31], and Ito et al. assessed arrival rSO_2_ in relation to neurological outcome rather than intra-arrest ROSC [41]. The quantitative evidence, therefore, supports study-specific signals and thresholds, but not generalized sensitivity, specificity, or cut-points that can be transferred unchanged across the full corpus [12,13,21,27,31,37,40,41].

The functional role of NIRS was also heterogeneous (Table A4). Across the included studies, NIRS was used as a prognostic marker, as a dynamic marker of evolving circulation, and as a CPR-quality feedback signal, and these roles frequently coexisted within the same study (Table A4). The strongest evidence supports NIRS as a dynamic marker of evolving circulation, as rising intra-arrest cerebral oxygenation was repeatedly associated with ROSC-related trajectories [13,14,36,37,38,39]. Evidence that NIRS can reliably anticipate ROSC before circulation is restored remains more limited [14,34,36,37,40,41]. A study reported a sustained rise before ROSC [36], whereas other cohorts found that single early values were poor discriminators [34,37] or that more informative associations emerged only after aggregation across defined intra-arrest windows, at post-ROSC time points, or at hospital arrival [14,37,40,41]. This pattern supports interpreting NIRS more cautiously as a dynamic marker of evolving circulation and ROSC confirmation than as a validated early predictor [14,34,36,37,40,41].

Commercial cerebral NIRS outputs are mixed arterial–venous tissue oxygenation signals that are typically interpreted using manufacturer-specified arterial–venous weighting assumptions rather than direct measurements of cerebral blood flow [52,53]. Experimental work also shows that the physiologic arterial contribution is not fixed and may increase in hypoxemia, which limits one-to-one interpretation of any given rSO_2_ or TOI value [52,54]. During CPR, rises in cerebral oxygenation are therefore compatible with improved cerebral oxygen delivery, but they may also reflect altered oxygen extraction or shifts in arterial–venous weighting within the sampled tissue [44,52,53,54]. Interpretation is further complicated by extracranial contamination, because scalp hypoxia has been shown to reduce measured cerebral saturation across devices [55], and several included studies noted that the recorded signal was not exclusively cerebral or could be influenced by scalp or CSF contributions [20,34,49]. This non-specificity is also consistent with human data showing a positive but incomplete correlation between rSO_2_ and invasive brain tissue oxygen tension under changing ventilation conditions (Spearman r = 0.50) [56]. CPR-specific experimental work in this review likewise showed that rSO_2_ did not always track invasive perfusion or oxygenation measures in parallel: after adrenaline, CPP and PbtO_2_ increased while rSO_2_ remained unchanged in a hypothermic porcine model [44], and skull- versus skin-based measurements diverged during porcine CPR, with only skull-based values correlating with brain tissue oxygen tension [47]. Consequently, increases in rSO_2_ during CPR should be interpreted as physiologically relevant but not mechanistically specific, because they may reflect improved cerebral perfusion, altered oxygen extraction, or non-cerebral signal contributions rather than direct evidence of restored cerebral blood flow alone [44,47,52,53,54,55,56]. Peripheral pulse oximetry also cannot substitute for cerebral NIRS during CPR, because it depends on a reliable pulsatile peripheral signal and becomes inaccurate when perfusion is markedly reduced; in an experimental clinical study, acceptable pulse-oximetry bias was maintained only above a systolic blood pressure of 80 mmHg [57].

Across the current literature, three partially overlapping clinical applications can be distinguished: ROSC-related transition detection or confirmation, prognostication of survival or neurological outcome, and assessment of CPR quality or cerebral perfusion during resuscitation [14,21,27,31,34,36,37,38,39,40,41,43]. The evidence is most consistent for ROSC-related transition monitoring, because several studies described abrupt rises or sustained increases in cerebral oxygenation around ROSC [36,38,39] and one prehospital cohort also documented declines with re-arrest [38]. Evidence for true pre-ROSC prediction is more limited and remains study-specific, but dynamic or threshold-based predictive candidate signals were reported in several cohorts [21,27,36,39,40]. Prognostic applications were more often based on temporally aggregated intra-arrest measures, prespecified thresholds, post-ROSC intervals, or hospital-arrival values rather than on very early single readings [14,27,31,34,35,37,41]. By contrast, assessment of CPR quality or cerebral perfusion appears to place greater technical demands on the signal, because studies focused on compression rate, arterial pressure, or compression-linked hemodynamics relied either on modest associations with conventional saturation outputs [19,21,43] or on higher-bandwidth waveform-derived features [5,30]. These patterns suggest that current evidence better supports NIRS for trend monitoring and ROSC-related transition detection than for stand-alone prognostication or real-time CPR-quality feedback across platforms [14,21,27,36,37,38,39,43].

### 4.3. Clinical Translation, Feasibility, and Evidence Gaps

Regarding our third research question, evidence varies across arrest settings, patient groups, and study models. It is strongest in adult OHCA within prehospital or emergency department resuscitation but remains sparse in pediatric cohorts, in-hospital settings, and longer-term outcome studies (Table A1 and Table A4).

Clinical application varied by context. In adult OHCA, studies mostly examined ROSC-associated trajectories, early-window monitoring, or arrival-based prognostication [14,34,36,37,40,41]. Evidence in pediatric populations was more limited. Two pediatric cohorts linked higher intra-arrest cerebral saturation with ROSC [31,35]. One multicenter cohort found that higher crSO_2_, increased time above threshold, and better survival and neurological outcomes were associated [31]. Another pediatric OHCA report described cerebral oximetry with a blood volume index as feasible decision support when end-tidal CO_2_ changes were lost during resuscitation [45]. Adult in-hospital evidence was less extensive but included higher maximum rSO_2_ among survivors in an ICU cohort [46], and CPR-time rSO_2_ as a stronger predictor of ROSC than ETCO_2_, with higher rSO_2_ also associated with 30-day survival in an IHCA cohort [27]. In an adult OHCA emergency department cohort, rSO_2_ showed a mild but significant association with mean arterial pressure during CPR [43]. Animal studies provided mechanistic and intervention data. These included site-dependent correlations with brain tissue oxygenation [47], oxygen-fraction effects on rSO_2_ or PbtO_2_ [48,49], etiologic differences in TOI trajectories [50], and abrupt ROSC-related signal changes moderately correlated between rSO_2_ and cardiac output in a pediatric swine model [23].

Ultimately, the main barriers to clinical translation are operational deployment constraints, selective or delayed monitoring, cross-platform non-comparability, and limited evidence that acting on NIRS changes clinical outcomes [7,22,31,36,40]. Feasibility studies identified a need for more personnel or training. Delays also arose from competing resuscitation priorities. Additional obstacles included limited space or power supply, probe shortages, and sensor issues [7,14,21,36,38,41,56]. Notably, several cohorts started NIRS only after CPR began or only for patients who stayed in arrest long enough for monitoring. This may under-represent brief arrests and early ROSC [35,36,41]. Furthermore, interpretation is limited by non-uniform proprietary algorithms and possible extracerebral contamination [7,34,40]. Associations with proposed CPR-quality surrogates were variable and context dependent, not uniformly strong [19,21,43,44]. In some studies, NIRS values were masked from treating teams or not used for real-time decision-making [29,35]. Finally, the only identified rSO_2_-guided workflow study did not demonstrate a clear ROSC advantage in the main IPTW analysis [22].

### 4.4. Limitations

The search was restricted to peer-reviewed English-language full texts indexed in Embase, MEDLINE, PubMed, and CINAHL; conference abstracts and proceedings were excluded, which may have limited capture of early engineering reports and device-validation studies. Restricting eligibility to conventional CPR and porcine experimental models improved agreement with the review objectives, yet limits generalizability to extracorporeal modalities and non-porcine experimental systems.

The review also reflects inherent restrictions of the literature itself. Notably, many studies lacked consistent reporting of acquisition and processing parameters, such as wavelengths, channel configuration, sampling rate, and handling of superficial signals, resulting in limited ability to interpret absolute values, compare device behaviours, and identify optimal workflows for reliable deployment. Moreover, between-platform differences in output types and device-specific processing assumptions prevented direct transfer of absolute thresholds across systems, constraining synthesis of prognostic cut-points in the literature (Table A2 and Table A3; [7,31,36,40]). Quantitative performance metrics were inconsistently reported, varying by signal family, timing window, outcome, and clinical context [12,13,21,27,31,37,40,41]. As a result, thresholds and sensitivity/specificity estimates were necessitated to be summarized narratively, rather than pooled, underscoring further synthesis challenges.

Furthermore, the evidence base is concentrated in adult OHCA and short-term ROSC-focused analyses, with comparatively less evidence in pediatric resuscitation, exclusively in-hospital settings, and longer-term survival or neurological outcomes (Table A1 and Table A4). Common study limitations included small-sample pilot designs, device-comparability concerns, incomplete capture of CPR-quality timing or related covariates, lack of clinician blinding in research-only designs, and signal-quality or artifact problems (Table A4). Since this was a scoping review, a formal risk-of-bias assessment was not undertaken. Therefore, differences in study quality may influence the mapped patterns, but these were not graded. Additionally, some charted variables relied on condensed narrative descriptions, which may obscure distinctions in timing windows, analytic methods, subgroup definitions, or resuscitation context. These patterns collectively frame the interpretive boundaries of the review.

### 4.5. Future Directions

Future development of NIRS for CPR will depend on shifting from predominantly retrospective association analyses toward approaches that can deliver interpretable, action-linked physiologic feedback during ongoing resuscitation. Currently, most CPR-process monitoring studies relate established vendor outputs (primarily rSO_2_-family indices and, in some studies, TOI) to arterial pressure, compression rate, or compression modality [9,19,21,43]. To build on this, future research should explicitly distinguish between conventional saturation measures and CPR-specific waveform-derived features, testing whether waveform-derived features offer additional information beyond standard saturation trends within the same events [5,30]. Multimodal study designs pairing NIRS with established CPR-quality indicators, such as arterial pressure [19,43] and ETCO_2_ [19,27], could provide a pragmatic framework for validating NIRS-derived features and clarifying the circumstances wherein cerebral oximetry diverges from other perfusion surrogates. As existing quantitative findings show that threshold performance varies by timing window, output family, and outcome [12,13,21,27,31,37,40,41], future quantitative studies should therefore prespecify the intended functional role of the signal, define the timing window used for analysis, and report performance measures more consistently to enable meaningful comparison of study-level thresholds across the literature. Addressing these needs will be crucial, particularly in underrepresented clinical contexts such as pediatric resuscitation, adult IHCA, and studies focused on survival and neurological outcomes rather than ROSC alone. While feasibility studies remain important, prospective interventional studies are also needed to determine whether NIRS-guided strategies alter care or outcomes.

If successful, this evolution may reposition NIRS from a primarily observational intra-arrest correlate to a more clinically actionable physiologic feedback modality, tailored to the dynamics of resuscitation.

## 5. Conclusions

This scoping review demonstrates that NIRS during conventional CPR has been studied primarily as a cerebral oxygenation monitor, with consistent intra-arrest associations between rising oxygenation patterns and return of spontaneous circulation (ROSC). Proposed thresholds varied by output family, timing window, device context, and outcome, and current evidence, therefore, does not support device-agnostic cut points or the use of NIRS as a stand-alone prognostic or termination tool. Most investigations rely on established vendor-derived saturation metrics acquired at low sampling frequencies, limiting evaluation of compression-synchronous or perfusion-responsive optical features. As a result, the literature supports NIRS as a feasible adjunct physiologic signal during CPR, but it is not yet a validated decision-guidance modality. Progress toward a clinically actionable application will require more complete acquisition reporting, direct platform comparison, and focused prospective development of CPR-specific optical metrics that reflect real-time perfusion dynamics.

## Data Availability

Data are contained within the article.

## Abbreviations

The following abbreviations are used in this manuscript:

ABG	Arterial blood gas
ALS	Advanced life support
AUC	Area under the curve
AVP	Arginine vasopressin
BLS	Basic life support
BVI	Blood volume index
CICU	Cardiac intensive care unit
CPC	Cerebral Performance Category
CPP	Cerebral perfusion pressure
CPR	Cardiopulmonary resuscitation
crSo_2_	Cerebral regional oxygen saturation
CSF	Cerebrospinal fluid
DBP	Diastolic blood pressure
ECPR	Extracorporeal cardiopulmonary resuscitation
ED	Emergency department
EMD	Electromechanical dissociation
EMS	Emergency medical services
ETCO_2_	End-tidal carbon dioxide
FiO_2_	Fraction of inspired oxygen
HEMS	Helicopter emergency medical services
HHb	Deoxygenated hemoglobin
HUP-CPR	Head-up cardiopulmonary resuscitation
ICU	Intensive care unit
IHCA	In-hospital cardiac arrest
IPTW	Inverse probability of treatment weighting
JBI	Joanna Briggs Institute
MAP	Mean arterial pressure
MCCD	Mechanical chest compression device
NIRS	Near-infrared spectroscopy
OHCA	Out-of-hospital cardiac arrest
O_2_Hb	Oxygenated hemoglobin
PaCO_2_	Partial pressure of arterial carbon dioxide
PaO_2_	Partial pressure of arterial oxygen
PbtO_2_	Partial pressure of brain tissue oxygen
PCC	Population–Concept–Context
PEA	Pulseless electrical activity
PED	Pediatric emergency department
PICU	Pediatric intensive care unit
POHCA	Pediatric out-of-hospital cardiac arrest
ROSC	Return of spontaneous circulation
rSO_2_	Regional cerebral oxygen saturation
SctO_2_	Cerebral tissue oxygen saturation
ScvO_2_	Central venous oxygen saturation
StO_2_	Tissue oxygen saturation
SWT	Stationary wavelet transform
THb	Total hemoglobin
THI	Tissue hemoglobin index
TOI	Tissue oxygenation index
VF	Ventricular fibrillation
VT	Ventricular tachycardia
ΔcHb	Change in total hemoglobin
ΔHHb	Change in deoxygenated hemoglobin
ΔO_2_Hb	Change in oxygenated hemoglobin
ΔTOI	Change in tissue oxygenation index

## Appendix A

**Table A1:** Study-level characteristics.

Article First Author/Year	Study Design	Optical Application	Setting	Episode Type	Witnessed	By Stander CPR	Initial Rhythm	Population	Age (Years)	Sex Male (%)	Sample Size
Francoeur 2022 [35]	Prospective single-centre observational pilot study	Monitoring; Prognostication (research only)	In-hospital (PICU/CICU/ED)	IHCA; OHCA	Not reported	Not reported	Ventricular Fibrillation (VF), pulseless Ventricular Tachycardia (VT), Asystole, Pulseless electrical activity (PEA)	Pediatric	Median 1.67 (0.42–14)	42.9	23
Shin 2022 [14]	Prospective observational cohort study	Prognostication	Prehospital	OHCA	49%	67%	Asystole, PEA, VF/VT	Adult	Mean 64 (SD 16)	56	59
Nelskylä 2023 [49]	Experimental animal study	Monitoring (research only)	Animal laboratory	Not applicable (experimental model)	Not applicable	Not applicable	shockable (induced VF)	Animal	14–18-week landrace	Not reported	28
Tsukuda 2021 [21]	Prospective observational cohort pilot study	Monitoring; Prognostication	Prehospital (ambulance transport)	OHCA	61.5% (ROSC); 16.4 (non-ROSC)	38.5% (ROSC); 42.9% (non-ROSC)	Shockable	Adult	Mean 72.4 (ROSC); 80.8 (non-ROSC)	73.1% (ROSC); 57.1% (non-ROSC)	81
Yazar 2019 [46]	Observational preliminary study	Monitoring; Prognostication	In-hospital (ICU)	IHCA	Not reported	Not reported	Not reported	Adult	Mean age 72.6 ± 4.2 (survivors); 77.3 ± 6.5 (no survivors)	40	20
Nelskylä 2017 [48]	Randomized experimental animal study	Monitoring	Animal laboratory	Not applicable (experimental model)	Not applicable	Not applicable	Shockable	Animal	Not reported	Not reported	19
Kishihara 2022 [43]	Prospective observational cohort study	Real-time guidance (quality indicator for chest compressions)	ED	OHCA	70.3%	32.4% yes	VF/pulseless VT, PEA, asystole	Adult	Median 75 (69–82)	83.8	37
Nelskylä 2022 [19]	Prospective observational cohort study	Monitoring	Prehospital (physician-staffed helicopter service)	OHCA	Not reported	71%	Shockable; non-shockable	Adult	Median 68 (59–73)	73	75
Takegawa 2021 [22]	Prospective multicentre interventional study with historical control cohort	Real-time guidance (rSO_2_-guided continuous chest compressions without rhythm checks)	ED	OHCA	42% (94)	55% (123)	Non-shockable (PEA and asystole)	Adult	Median 77 (70–83)	57	225
Schewe 2014 [38]	Prospective observational feasibility cohort study	Monitoring (research only)	Prehospital	OHCA	Not reported	Not reported	Shockable (VF), non-shockable (asystole)	Adult	Mean 73 ± 13	80	10
Baloglu Kaya 2021 [12]	Prospective randomized clinical trial	Monitoring; Prognostication	ED	IHCA	100%	Not reported	Asystole, PEA, VF/pulseless VT	Adult	MCCD: 71.85 ± 13.46; Manual: 71.37 ± 13.46	MCCD: 62.5; Manual: 62.9	75
Storm 2016 [34]	Prospective observational cohort study	Prognostication (research only)	Prehospital; in-hospital	OHCA	Not reported	Not reported	VF/Asystole/EMD	Adult	Mean 61 (non- ROSC), 66 (pre-ROSC), 68 (post ROSC)	Post ROSC 100%; Pre ROSC 80%; No ROSC 78%	29
Tsukuda 2019 [40]	Prospective observational cohort study	Prognostication; Real-time guidance	ED	OHCA	51.3%	40.2% yes	Shockable 11.1%	Adult	Mean 69.7 ± 17.3	57.3	117
Asim 2014 [42]	Observational cohort/Not reported	Monitoring; Prognostication	ED	OHCA	No	Not reported	VF, Asystole, PEA	Adult	Mean 64.09 ± 13.66	47.8	23
Singer 2015 [13]	Retrospective observational study	Monitoring; Prognostication	ED	OHCA	Not reported	Not reported	VF/VT, PEA, asystole	Adult	Mean 68.7 ± 14.9	84.70%	59
Kalkan 2015 [25]	observational study	Prognostication	ED	OHCA	No	Not reported	VF, PEA, asystole	Adult	Mean 63.06 ± 11.66	50%	34
Meex 2013 [7]	Observational feasibility study	Monitoring (research only)	Prehospital; in-hospital	OHCA; IHCA	Not reported	Not reported	Not reported	Adult	Mean 66 ± 20	71.40%	14
Prosen 2018 [36]	Prospective observational study	Monitoring (research only)	Prehospital	OHCA	90% (ROSC); 90% (no ROSC)	5% (ROSC); 13% (no ROSC)	VF/VT, PEA, asystole	Adult	Mean age 65; Median 70.5 (ROSC) vs. 67.0 (non-ROSC)	84% (overall); 81% (ROSC), 87% (no ROSC)	53
Genbrugge 2018 [39]	Prospective non-randomized multicenter study	Monitoring; Prognostication	Prehospital	OHCA	74% (ROSC); 51% (no ROSC)	41% (ROSC); 39% (no ROSC)	Asystole, VF, PEA	Adult	Mean 68 ± 14 (ROSC) vs. 69 ± 15 (non-ROSC)	65% (ROSC); 73% (no ROSC)	329
Al-Subu 2020 [23]	Prospective experimental animal study	Monitoring	Animal laboratory	Not applicable (experimental model)	Not applicable	Not applicable	Shockable (VF)	Animal	2–3 months	Not reported	8 swine, 28 VF arrests
Duvekot 2015 [58]	Prospective single-centre observational cohort study	Prognostication (research only)	ED	OHCA	Not reported	Not reported	VF 57%	Adult	Mean 64	63	46
Frisch 2012 [29]	Case series	Monitoring; Real-time guidance (research only)	Prehospital	OHCA	3 of 5 cases	100%	Not reported	Adult	84, 76, 81, 83, 61	20	5
Deakin 2016 [51]	Prospective cohort study	Research only	In-hospital	IHCA	Yes	Not reported	Asystole, PEA, VF/VT	Adult	Median 77	58.30%	36
Putowski 2025 [27]	Prospective single-centre observational cohort study	Prognostication; Monitoring	In-hospital	IHCA	Not reported	Not reported	Asystole, PEA, VF/VT	Adult	Mean 68 (SD 12)	63.5	104
Sanz-Pescador 2024 [30]	Observational cohort study	Real-time guidance (research only)	Prehospital	OHCA	Not reported	Not reported	Not reported	Adult	Not reported	Not reported	30
Pourzand 2024 [26]	Randomized pre-clinical experimental animal study	Monitoring	Animal research laboratory	Not applicable (experimental model)	Not applicable	Not applicable	Shockable (VF)	Animal	Not reported	Not reported	22
Raymond 2024 [31]	Multicenter observational study	Prognostication	In-hospital	IHCA	Not reported	Not reported	Not reported	Pediatric	Median 0.3 (0.1–1.4)	56	123
Koyama 2023 [50]	Experimental animal study	Monitoring	Animal research laboratory	Not applicable (experimental model)	Not applicable	Not applicable	VF-CA; A-CA	Animal	Median 67.5 days (VF-CA group); 76.0 days (A-CA group)	0	20
Jang 2023 [37]	Single-center observational cohort	Prognostication	Prehospital to ED	OHCA	84.6% (ROSC); 43.6% (non-ROSC)	61.5% (ROSC); 48.7% (non-ROSC)	Shockable	Adult	Median 55 (ROSC); 72 (non-ROSC)	84.6% (ROSC); 59.0% (non-ROSC)	52
Košir 2023 [24]	Single-center observational cohort	Monitoring; Prognostication	Prehospital	OHCA	50% of cases	70% yes	VF, asystole, PEA	Adult	Median 66.0 (60.5–79.5)	65	20
Koyama 2013 [5]	Non-consecutive observational case series	Monitoring; Prognostication; Real-time guidance	ED	OHCA	Not reported	Not reported	VF, PEA, Asystole	Adult	Mean 79 (55–99)	66	15
Parnia 2012 [10]	Feasibility (pilot study)	Prognostication	In-hospital	IHCA	Not reported	Not reported	VF, Asystole, PEA	Adult	Mean 76 ± 15 (survivors); 73 ± 11 (non-survivors)	Not reported	19
Bein 2006 [47]	Experimental animal study	Monitoring	Animal research laboratory	Not applicable (experimental model)	Not applicable	Not applicable	VF	Animal	12 to 16 weeks	Not reported	12
Bouček 2018 [9]	Prospective experimental animal study	Monitoring	Animal research laboratory	Not applicable (experimental model)	Not applicable	Not applicable	VF	Animal	16–20 weeks	0% (all female)	24
Putzer 2016 [44]	Experimental animal study	Monitoring	Animal research laboratory	Not applicable (experimental model)	Not applicable	Not applicable	VF	Animal	12 to 16 weeks	Not reported	9
Lennmyr 2010 [28]	Experimental animal study	Monitoring	Animal research laboratory	Not applicable (experimental model)	Not applicable	Not applicable	shockable (VF)	Animal	Not reported	Not reported	17
Abramo 2014 [45]	Case series	Monitoring; Prognostication; Real-time guidance;	ED (pediatric emergency department)	OHCA	Not reported	yes	Case 1: VF; Case 2: asystole, VF	Pediatric	Case 1: 15-year-old; Case 2: 14-year-old	Case 1 male; Case 2 female	2
Kim 2022 [20]	Prospective interventional pilot study	Research only	ED	OHCA	50% of cases	53.6	Shockable 10.7%	Adult	80.5 (71.5–84.0)	57.10%	28
Ito 2014 [41]	Prospective multicenter observational cohort study	Prognostication	ED	OHCA	53% of cases	39	VF/VT, Asystole, PEA	Adult	Mean 71	60%	672

**Table A2:** NIRS device specifications and acquisition settings.

First Author/Year	Brand/Model	Wavelengths (nm)	Number of Sensors	Sampling Rate (Hz)	Wired/Wireless	Sensor Site
Francoeur 2022 [35]	Equanox 7600 (Nonin Medical, Plymouth, MN, USA)	Not reported	≥1	0.25	Wired	Cerebral (forehead)
Shin 2022 [14]	SenSmart Model X-100 Universal Oximetry System (Nonin Medical, Inc.)	Not reported	1	Not reported	Wired	Cerebral (left forehead)
Nelskylä 2023 [49]	INVOS 5100C Cerebral Oximeter (Somanetics Inc., Troy, MI, USA)	Not reported	Not reported	Not reported	Wired	Cerebral (left forehead)
Tsukuda 2021 [21]	CCR-1 (Hamamatsu Photonics, Hamamatsu-City, Shizuoka, Japan)	Not reported	1	Not reported	Wired	Cerebral forehead left-lateral above eyebrow
Yazar 2019 [46]	INVOS 5100C (Somanetics, Troy, MI, USA)	Not reported	2	Not reported	Wired	Cerebral, bilateral forehead
Nelskylä 2017 [48]	INVOS 5100C (Somanetics Inc., Troy, MI, USA)	Not reported	1	Not reported	Wired	Cerebral left forehead
Kishihara 2022 [43]	INVOS 5100C (Medtronic, Boulder, CO, USA)	Not reported	2	Not reported	Wired	Cerebral left and right forehead
Nelskylä 2022 [19]	SenSmart X-100 (Nonin Inc., Plymouth, MN, USA)	Not reported	2	0.25	Wired	Cerebral, bilateral forehead
Takegawa 2021 [22]	TOS-QQ® brain oximeter (TOSTEC Co., Ltd., Tokyo, Japan)	Not reported	Not reported	Not reported	Not reported	Cerebral forehead
Schewe 2014 [38]	Equanox Model 7600 (Nonin Medical, Plymouth, MN, USA)	Not reported	1	0.25	Wired	The left frontal forehead lateral to midline and above the eyebrow
Baloglu Kaya 2021 [12]	Root and O3™ Regional Oximeter (Massimo, Irvine, CA, USA)	Not reported	2	0.5	Wired	Cerebral, bilateral forehead
Storm 2016 [34]	INVOS 5100C (Covidien; Mansfield, MA, USA)	724, 810	2	Not reported	Wired	Cerebral, bilateral forehead
Tsukuda 2019 [40]	NIRO-200NX (Hamamatsu Photonics, Hamamatsu-City, Shizuoka, Japan)	Not reported	2	Not reported	Wired	Cerebral bilateral supraorbital
Asim 2014 [42]	INVOS 5100C (Covidien, Boulder, CO, USA)	Not reported	2	Not reported	Wired	Cerebral; bilateral frontal
Singer 2015 [13]	Equanox Advance monitor (Nonin Medical Inc., Plymouth, MN, USA)	Not reported	1	0.25	Wired	Cerebral forehead
Kalkan 2015 [25]	INVOS 5100C (Covidien, Boulder, CO, USA)	Not reported	4	Not reported	Wired	Bilateral cerebral; bilateral abdominal
Meex 2013 [7]	FORE-SIGHT monitor (CAS Medical Systems, Branford, CT, USA); Equanox Advance monitor (Nonin Medical Inc., Plymouth, MN, USA)	Not reported	2 (FORE-SIGHT); 1 (Equanox)	Not reported	Wired	FORE-SIGHT bilateral forehead; Equanox unilateral forehead (right forehead)
Prosen 2018 [36]	INVOS Oximeter (Somanetics Corporation, Troy, MI, USA)	Not reported	2	Not reported	Wired	Cerebral, bilateral forehead
Genbrugge 2018 [39]	Equanox 7600 or SenSmart X-100 (Nonin Medical Inc., Plymouth, MN, USA)	Not reported	1	0.25	Wired	Right forehead
Al-Subu 2020 [23]	Somatic INVOS 5100C (Somanetics Corpora-tion, Troy, MI, USA)	Not reported	2	Not reported	Wired	Left forehead (cerebral) and left flank over kidney (somatic/renal)
Duvekot 2015 [58]	NIRS (Covidien, The Netherlands)	Not reported	1	Not reported	Not reported	Cerebral (forehead)
Frisch 2012 [29]	InSpectra StO_2_ Tissue Oxygenation Monitor (Hutchinson Technology, Hutchinson, MN, USA)	Not reported	1	Not reported	Wired	Thenar eminence (hand)
Deakin 2016 [51]	Equanox Model 7600 (Nonin Medical, Plymouth, MN, USA)	Not reported	1	1 per 6 s	Wired	Cerebral (forehead)
Putowski 2025 [27]	Masimo Open Connect (MOC-9) (Masimo, Irvine, CA, USA)	Not reported	Not reported	0.5	Wired	Cerebral
Sanz-Pescador 2024 [30]	NIRO-200NX (Hamamatsu Photonics, Hamamatsu, Japan)	Not reported	1	20	Wired	Cerebral (left frontal lobe)
Pourzand 2024 [26]	SenSmart X-100 (Nonin Medical Inc., Plymouth, MN, USA)	Not reported	Not reported	Not reported	Wired	Cerebral
Raymond 2024 [31]	INVOS 5100C (Medtronic, Minneapolis, MN, USA); Equanox 7600 (Nonin Medical, Plymouth, MN, USA)	Not reported	1 (INVOS); 1 (Equanox)	0.25 (INVOS); 1 (Equanox)	Wired	Cerebral (single sensor on either forehead)
Koyama 2023 [50]	NIRO 200NX (Hamamatsu Photonics, Hamamatsu-shi, Shizuoka, Japan)	Not reported	2	Not reported	Wired	Cerebral bilateral anterior to coronal suture
Jang 2023 [37]	INVOS 5100C (Covidien, Boulder, CO, USA)	Not reported	2	Not reported	Wired	Cerebral, bilateral forehead
Košir 2023 [24]	SenSmart X-100 (Nonin Medical, Inc., Playmouth, MN, USA)	Four wavelengths	2	0.25	Wired	Cerebral right forehead and somatic right thenar
Koyama 2013 [5]	NIRO (Hamamatsu Photonics, Hamamatsu-shi, Shizuoka, Japan)	700–1300	2	0.5 recalibrated to 20	Wired	Cerebral, bilateral forehead
Parnia 2012 [10]	Invos (Somanetics, Troy, MI, USA)	Not reported	2	Not reported	Wired	Cerebral (left or right forehead)
Bein 2006 [47]	NIRO 300 (Hamamatsu Photonics, Hamamatsu City, Japan)	775, 810, 850, 910	2	0.2	Wired	Cerebral forehead
Bouček 2018 [9]	INVOS 5100C (Medtronic Inc., Minneapolis, MN, USA)	650–900	2	Not reported	Wired	Two INVOS infant-neonatal probes; cerebral probe on forehead (middle of eyes, over cerebrum); peripheral probe on left thigh (over a muscle)
Putzer 2016 [44]	INVOSs (Somanetics Inc., Troy, MI, USA)	Not reported	1	Not reported	Wired	Cerebral, right forehead
Lennmyr 2010 [28]	INVOS (Somanetics Inc., Troy, MI, USA)	Not reported	1	Not reported	Wired	parietal skull
Abramo 2014 [45]	NIR INVOS 5100v (Somanetics Inc., Troy, MI, USA)	Not reported	2	0.2	Wired	Cerebral, bilateral forehead
Kim 2022 [20]	NIRSIT ON (OBELAB Inc., Seoul, Republic of Korea)	780, 850	2	32.552	Wireless	Cerebral, bilateral forehead
Ito 2014 [41]	INVOS 5100C (Covidien, Boulder, CO, USA)	730, 805	2	Not reported	Wired	Cerebral, bilateral forehead

**Table A3:** Processing, synchronization, and study context variables.

First Author/Year	Metric Family	Timing Window	Compression Context	Compression Ratio	Time Alignment Method	Artifact Handling Methods	Data Loss (%)	Software/Toolbox	Exposure of Interest/NIRS Feature
Francoeur 2022 [35]	rSO_2_	During CPR	Not reported	Not reported	Not reported	Manual artifact removal plus noise reduction filters.	Not reported	MATLAB custom for DBP; REDCap	Mean/median value
Shin 2022 [14]	rSO_2_	During compressions; pre-ROSC; Early post ROSC ≤ 5 min	Not reported	Not reported	Synchronized via defibrillator time stamps	Not reported	Not reported	Not specified	Initial value; Mean/median value
Nelskylä 2023 [49]	rSO_2_	during pre-arrest; untreated VF; 5 min of ineffective manual chest compressions; mechanical CPR; after ROSC.	Manual and Mechanical	Poor CPR: 82/min (FiO_2_ 50%); 79/min (FiO_2_ 100%); Mechanical CPR: 101/min	Narrative	Not reported	n = 1 (50% group); n = 1 (100% group)	MPR2 logO Dat-alogger, SPSS 27.0.1.0	Mean/median value; Change/trajectory (Δ)
Tsukuda 2021 [21]	TOI; O_2_Hb; HHb	During pre-hospital CPR, ambulance transportation, initial TOI at probe attachment in ambulance, final TOI at arrival to ED	Manual	Not reported	Not reported	Not reported	Not reported	SPSS; R	Change/trajectory (Δ)
Yazar 2019 [46]	rSO_2_	During compressions; Early post ROSC ≤ 10 min	Manual	Not reported	Not reported	Not reported	Not reported	SPSS (analysis)	Mean/median value; Maximum value;
Nelskylä 2017 [48]	rSO_2_	Pre ROSC (VF period prior to CPR); during compressions (CPR); early post ROSC (20 min after ROSC)	Mechanical chest compressions (LUCAS); ventilation by manual bag valve ventilation	Manual bag valve ventilation at 10/min	Synchronized to invasive PbO_2_	Not reported	Not reported	Not reported	Mean/median value; Change/trajectory (Δ)
Kishihara 2022 [43]	rSO_2_	During compressions, 0 to 15 min after arrival	Not reported	Not reported	Narrative only (measurements recorded at predefined minute marks: 0, 3, 6, 9, 12, 15 min)	Not reported	n = 4	EZR version 1.38; R version 3.5.2; SAS version 9.4 (analyses)	Median value; log-transformed value
Nelskylä 2022 [19]	rSO_2_	During CPR compressions	Manual and mechanical (LUCAS) continuous with supraglottic airway	Continuous	Device and defibrillator clocks synchronized weekly	Not reported	n = 4	Prism 9.0 (analysis)	median value; Change/trajectory (Δ)
Takegawa 2021 [22]	rSO_2_	During compressions: Continuous CC up to 16 min or until rSO_2_ target achieved	Manual and mechanical	Continuous	Not reported	Not reported	Not reported	R software version 3.6.3 (statistical analysis)	Mean/median/max value; baseline value
Schewe 2014 [38]	rSO_2_	During CPR	Manual vs. Mechanical	Not reported	Device time stamps and manual event markers for ROSC, use of mechanical compression device, and termination of CPR	Not reported	10.2	Microsoft Excel	Change/trajectory (Δ); waveform features
Baloglu Kaya 2021 [12]	rSO_2_	During compressions	Manual vs. Mechanical (LUCAS2)	Not reported	Not reported	The higher one of the two rSO_2_ values was used for analysis.	Not reported	IBM SPSS Statistics 21.0; MedCalc 19	Mean/median value
Storm 2016 [34]	rSO_2_	During compressions; near ROSC (within 2 min after ROSC)	Not reported	Not reported	Narrative (during CPR or within 2 min post ROSC)	None reported	Not reported	INVOS Analytics Tool, version 1.2	Initial value
Tsukuda 2019 [40]	TOI	During compressions	Manual	Not reported	Narrative	Not reported	n = 21	SPSS (V. 25) and R statistical software (V. 1.0.143)	Initial value
Asim 2014 [42]	ScO2	During compressions	Manual	Not reported	Narrative	Not reported	Not reported	SPSS 18.00	Change/trajectory (Δ)
Singer 2015 [13]	rSO_2_	during CPR	Manual and mechanical	Not reported	Narrative (from time sensor placed to ROSC or CPR termination)	Artifact values recognized by absent value or values at least three SDs away from mean rSO_2._	Not reported	Not reported	Mean/median value
Kalkan 2015 [25]	ScO2; rSO_2_	during CPR	Manual	Not reported	Not reported	Not reported	Not reported	SPSS 18.00 (statistical analysis)	Change/trajectory (Δ)
Meex 2013 [7]	SctO2	during CPR; at/near ROSC	Not reported	Not reported	Not reported	Protective adhesive tape used to minimize external light interference.	Not reported	SPSS V19.0	Initial/Maximum value; Change/trajectory (Δ)
Prosen 2018 [36]	rSO_2_	during CPR; at/near ROSC; early post-ROSC	Manual	Not reported	Defibrillator timestamps	Not reported	8.62	SigmaPlot software (Stat Co, Version 11.0)	Initial value; Change/trajectory (Δ)
Genbrugge 2018 [39]	rSO_2_	During compressions (prehospital ALS); pre-ROSC; at/near ROSC	manual or mechanical (LUCAS/AutoPulse)	Not reported	Event button; ROSC time noted on Utstein forms	Not reported	12.71	SPSS 22.00; GraphPad Prism 5.01 (data downloaded per manufacturer instructions)	Change/trajectory (Δ)
Al-Subu 2020 [23]	rSO_2_	Pre-arrest; during CPR; after ROSC	Manual	~100/min	Not reported	Not reported	Not reported	SAS	Change/trajectory (Δ)
Duvekot 2015 [58]	TOI	During compressions	Not reported	Not reported	Narrative	Not reported	Not reported	IBM SPSS 20	Initial value
Frisch 2012 [29]	StO_2_	During compressions; at/near ROSC; pre-ROSC; early post-ROSC during transport to hospital (exact duration not reported)	Not reported	Not reported	Narrative	Not reported	Not reported	Not reported software	Change/trajectory (Δ)
Deakin 2016 [51]	rSO_2_	During CPR	Not reported	Not reported	Event button on oximeter	Outlier removal (rSO_2_ values > 3 SD from mean removed)	n = 1	SAS version 9.3 (analysis); REDCap used for data entry	Initial value; Mean/median value; Change/trajectory (Δ)
Putowski 2025 [27]	rSO_2_	During compressions; near ROSC; post-ROSC < 24 h	Mechanical	Not reported	Not reported	Not reported	Maximum of 10% per patient	SPSS version 29.0; R; MedCalc	Mean/median/max value; Change/trajectory (Δ)
Sanz-Pescador 2024 [30]	∆cHb	During compressions (segments during CC series)	Not reported	Not reported	Thoracic impedance synchronized	Stationary wavelets transform (SWT); Kurtosis-based quality control	Not reported	MATLAB custom GUI	Waveform features
Pourzand 2024 [26]	rSO_2_	Measurements at baseline (pre-VF), end of 15 min of untreated VF, during CPR at 10 and 24 min, and at 15 and 60 min after ROSC (during early post ROSC ≤ 1 h)	Mechanical	105/min with 10:1 compression to ventilation ratio	Not reported	Not reported	Not reported	BioPac (acquisition only); SPSS version 26	Mean Value/Change/trajectory (Δ)
Raymond 2024 [31]	crSO_2_	During compressions (entire event); first 5 min; last 5 min	Not reported	Not reported	Not reported	Not reported	Not reported	R, R stats, and pROC package Version 4.2.2	Mean/median value
Koyama 2023 [50]	TOI	Before, during compressions; after CPR	Mechanical (LUCAS 2)	Asynchronous ventilation 10 breaths/min	Synchronized across monitors	Not reported	Not reported	EZR 1.52 on R	Median value; Change/trajectory (Δ)
Jang 2023 [37]	rSO_2_	During compressions; first 5 min; first 10 min; entire CPR	Manual	Not reported	Not reported	Not reported	Not reported	SPSS 18.0; Med-Calc 12.7.7.0	Initial value; Max/Min/Mean value; Change/trajectory (Δ)
Košir 2023 [24]	rSO_2_	During compressions	Not reported	Not reported	Narrative (paired with intervention protocol).	Spiking signals removed as artifacts.	n = 5	SenSmart v1.0.1.0 (Nonin); MedCalc ver. 20.104	Initial value; Maximum value; Change/trajectory (Δ); End-CPR value
Koyama 2013 [5]	ΔcHb; TOI	During compressions	Manual	Not reported	Not reported	Not reported	Not reported	Not reported	waveform features; Change/trajectory
Parnia 2012 [10]	rSO_2_	During compressions	Not reported	Not reported	Not reported	Not reported	n = 4	PRISM 6	Mean value; Change/trajectory (Δ)
Bein 2006 [47]	TOI; THI	Pre ROSC (VF then CPR) and during compressions; at/near ROSC and early post ROSC (followed for 1 h after resuscitation)	Not reported	100/min	Narrative	Values were aver-aged over 30 s.	Not reported	SPSS	Change/trajectory (Δ)
Bouček 2018 [9]	rSO_2_	During compressions (five minutes of CPR); comparisons used mean rSO_2_ over 15 min baseline, 3rd minute of untreated CA, and five minutes of CPR	Mechanical compressions (LUCAS 2)	Not reported	Narrative (baseline vs. untreated CA vs. CPR periods)	Not reported	<10%	MedCalc 18	Mean/median value; Change/trajectory (Δ)
Putzer 2016 [44]	rSO_2_	During compressions, includes periods before and after a CPR interruption and after adrenaline administration during ongoing CPR	Mechanical chest compression (LUCAS2TM)	Not reported	Protocol-defined time points relative to CA/CPR phases and adrenaline administration	Not reported	Not reported	SPSS 20	Mean value; Change/trajectory (Δ)
Lennmyr 2010 [28]	rSO_2_	During resuscitation	Mechanical chest compressions (LUCAS)	100 cpm	Narrative/structured timepoints	Not reported	Not reported	Workbench 3.0 (Strawberry Tree Inc.) for acquisition; Microsoft Excel 2007 and Prism 4.0 for statistics	Trajectory
Abramo 2014 [45]	rSO_2_; Blood Volume Index (BVI)	Case 1 during chest compressions; Case 2 postarrest in PED	Not reported	Not reported	Not reported	Not reported	Not reported	Not reported	Not reported
Kim 2022 [20]	O_2_Hb; HHb; THb;	During compressions	Mechanical	Not reported	Narrative	Removed detached/zero/spiking segments; excluded recordings with >70% poor-quality data.	14.28	R-package software, version 4.0.5	Maximum value; Change/trajectory (Δ)
Ito 2014 [41]	rSO_2_	During resuscitation upon hospital arrival	Not reported	Not reported	Not reported	Not reported	n = 1	JMP 10.0.0, MedCalc 12.3.0, STATA 11.1 (analysis)	Initial value; minimum value at hospital arrival

**Table A4:** Study goals, key findings, and limitations.

First Author Year	Aim	Primary Finding	Main Finding	Feasibility Notes	Notes/Limitations
Francoeur 2022 [35]	To examine whether higher rSO_2_ values recorded during arrest were linked to achieving ROSC and surviving to hospital discharge.	ROSC; Survival	During pediatric in-hospital cardiac arrest, higher intra-arrest cerebral rSO_2_, particularly higher median values and a greater proportion of time with rSO_2_ above 50% in the final five minutes of CPR, was associated with ROSC, but these rSO_2_ metrics did not translate into improved survival to hospital discharge.	The NIRS device was brought in by a respiratory therapist who was not directly involved in the resuscitation; at least one cerebral probe was placed quickly on the forehead, any probe already present for clinical care was left in place, and the monitor was positioned outside the resuscitation field and out of view of the CPR team.	The study enrolled a small convenience cohort and applied NIRS only to patients who remained in arrest long enough for probe placement during CPR, which likely under-sampled brief arrests in which ROSC occurred before monitoring could be initiated.
Shin 2022 [14]	To characterize early and ongoing rSO_2_ trajectories during first-responder resuscitation and evaluate how these patterns relate to ROSC and survival with favourable functional outcome.	ROSC; Survival; Neurological outcome	rSO_2_ was low at the onset of resuscitation (mean 41%) and rose during first-responder CPR; within the first five minutes, rSO_2_ values were higher in patients who subsequently achieved ROSC, and early post-ROSC rSO_2_ levels were higher among survivors with favourable neurological outcome (CPC 1–2).	EMS providers received 45 min of hands-on training; the sensor was applied to the left forehead after first-responder CPR had started, the screen was covered to blind EMS providers, and the initial three-person first-responder team was able to deploy the oximeter relatively early.	EMS providers were blinded to cerebral oximetry readings, so oximetry was not used to guide treatment decisions. The modest sample size limited more advanced inferential analyses, and early application may reduce generalizability.
Nelskylä 2023 [49]	To compare ventilation using 100% versus 50% oxygen during ineffective manual chest compressions and assess whether the higher oxygen fraction improves cerebral oxygenation.	Physiologic comparator; Oxygen-fraction effect	Comparing oxygen fractions during ineffective manual chest compressions, ventilation with 100% FiO_2_ increased brain tissue oxygen tension (PbtO_2_) relative to 50% FiO_2_ but did not meaningfully alter cerebral rSO_2_; during mechanical CPR, PbtO_2_ and rSO_2_ were similar with FiO_2_ 50% and 100%.	Not reported	The use of an experimental pig model under general anesthesia may not fully reflect human cardiac arrest, as the animals were a homogeneous group of young, healthy pigs, and the study was not blinded. NIRS measurements may also have been influenced by anatomical differences in skull and skin thickness, as well as by extracerebral vasoconstriction induced by adrenaline administration.
Tsukuda 2021 [21]	To examine the association between TOI and ROSC.	ROSC	During pre-hospital manual CPR for OHCA, larger increases in tissue oxygenation index (ΔTOI) during ambulance transport strongly predicted ROSC and were positively correlated with higher chest compression rates. ΔTOI thresholds in the range of approximately +5–8% discriminated episodes with ROSC, whereas decreases of ≤−2% were associated with non-ROSC.	Inside the ambulance, one of three trained paramedics applied the probe to the left forehead to minimize interruptions to CPR; the device was portable and battery-operated for two hours, but only five EMS teams were equipped. Apparatus dysfunction occurred in 19 of 104 patients (attachment failure or start-up delay), and 23 of 81 patients did not meet guideline chest compression rates due to the narrow ambulance space; paramedics were not educated on the meaning of NIRS values and did not adjust CPR based on TOI.	This was a single-center pilot with a small sample, and only five EMS teams were equipped with NIRS devices; numerous data errors were attributed to probe performance, and ΔTOI cut-offs were specific to this study. The NIRS device did not measure chest compression depth, and analyses were limited to mean compression rate and its correlation with ΔTOI, with limited neurological outcome data and could not evaluate the change in PaO_2_ during transport.
Yazar 2019 [46]	To assess how effectively chest compressions support cerebral oxygenation during ongoing CPR.	ROSC; Survival; Neurological outcome	Maximum rSO_2_ values during resuscitation were higher in patients who achieved ROSC than in non-survivors, and both the minimum and mean rSO_2_ during CPR were positively correlated with mean FOUR scores at one week among survivors.	Study-related procedures did not interfere with routine CPR practice.	The cohort was small, and the authors noted that technical monitoring in ICUs is complex, limiting the scope of the study’s conclusions. Additional illnesses may have influenced post-resuscitation FOUR scores, and the authors could not assess the effect of disease on FOUR.
Nelskylä 2017 [48]	To test whether administering 50% oxygen, compared with 100% oxygen, preserves cerebral oxygenation and reduces disruption of cardiac mitochondrial respiration during CPR.	Physiologic comparator; Oxygen-fraction effect	During CPR, FiO_2_ 50% resulted in lower cerebral rSO_2_ values on NIRS than FiO_2_ 100%, whereas brain tissue oxygen tension (PbO_2_) during CPR did not differ significantly between the two FiO_2_ groups.	The NIRS sensor was secured on the left forehead, and an invasive PbO_2_ probe was placed through a right-forehead burr hole and advanced to approximately 1 cm below the dura.	The study was unblinded and used young, healthy pigs under general anesthesia. Differences in forehead anatomy between pigs and humans may affect NIRS values.
Kishihara 2022 [43]	To analyze the relationship between mean arterial pressure and rSO_2_ during resuscitation and assess whether rSO_2_ reflects chest-compression quality.	Chest compression quality.	This analysis demonstrated a modest but statistically significant positive association between log-transformed rSO_2_ and log-transformed mean and systolic arterial pressures during resuscitation, leading the authors to propose cerebral rSO_2_ as a non-invasive indicator of chest compression quality.	Arterial pressure monitoring was described as requiring an arterial catheter and technical skill, whereas rSO_2_ was described as non-invasive.	The observed association was mild, which may limit clinical applicability for assessing chest compression quality. The study included only patients with poor prognosis, all of whom had a CPC of 5 at 90 days.
Nelskylä 2022 [19]	To estimate how frequently hyperoxia occurs during and immediately after successful CPR and to identify factors associated with intra-arrest cerebral oxygenation measured by NIRS.	Physiologic comparator; Hyperoxia association	In adult OHCA managed by a physician-staffed HEMS service, severe hyperoxia during or immediately after CPR was uncommon. Cerebral rSO_2_ measured by NIRS during CPR showed only a weak correlation with arterial blood pressure and no association with PaO_2_, PaCO_2_ or EtCO_2_, but increased after the initiation of mechanical chest compressions.	The study protocol was described as cumbersome and difficult to execute in time-critical prehospital conditions, requiring screening of many more patients than could be included. The insertion of invasive blood pressure lines and the collection of arterial blood gases during CPR were described as difficult, and the distinction between arterial and venous origin could not always be confirmed; invasive blood pressure values could not be obtained for all patients. The crew underwent theoretical and simulation training to support protocol compliance without compromising quality of care.	The sample may not reflect typical OHCA populations because delays to HEMS arrival and inclusion criteria restricted enrollment to patients still in cardiac arrest at HEMS arrival, contributing to a low secondary survival rate. Technical and logistical barriers limited invasive blood pressure and ABG measurements during CPR, and the authors noted that multiple technical issues with NIRS measurements could have influenced rSO_2_ values and may not have been fully captured.
Takegawa 2021 [22]	To assess the effectiveness of an rSO_2_-guided resuscitation strategy that omits rhythm checks, building on prior work.	ROSC; CPR guidance/decision support	In this pilot evaluation of TripleCPR, an rSO_2_-guided protocol using continuous chest compressions without routine rhythm checks, ROSC rates did not differ significantly from historical controls, and no serious adverse events were reported. The findings suggest that rSO_2_-guided protocols could be used to redesign the timing of rhythm checks, although this implementation did not improve ROSC.	The NIRS oximeter was attached within one minute of hospital arrival; chest compressions were briefly paused to check the tracheal tube position, ultrasonography was performed, and adrenaline was administered every four minutes.	The study was nonblinded and used a historical control cohort and did not evaluate neurological prognosis. Omitting rhythm checks every two minutes may have led to a missed conversion to a potentially shockable rhythm.
Schewe 2014 [38]	To evaluate whether NIRS monitoring is feasible as a surrogate measure of cerebral perfusion during physician-staffed out-of-hospital resuscitation.	ROSC; Feasibility	Prehospital NIRS monitoring during OHCA in a physician-staffed EMS setting was feasible, with rSO_2_ trajectories tracking clinical transitions: increases accompanied ROSC, whereas declines aligned with re-arrest. In that cohort, rSO_2_ values were higher during mechanical than manual chest compressions and were generally higher among patients who achieved ROSC.	Prehospital rSO_2_ monitoring achieved 89.8% valid recording time, with forehead optode placement in under 30 s by three briefly trained physicians and no interruption to basic life support.	This very small single-center feasibility cohort (10 OHCA patients, three with ROSC) ended monitoring at hospital arrival and used unilateral frontal sensing; ETCO_2_ was not stored and advanced artifact control was not implemented, limiting the strength of associations between rSO_2_, hemodynamics, and long-term outcomes.
Baloglu Kaya 2021 [12]	To compare rSO_2_ during manual versus mechanical chest compressions in witnessed ED cardiac arrests, and to examine how compression approach and perfusion relate to survival and neurologic outcomes.	ROSC	In the comparison of mechanical chest compression devices (MCCD) and manual CPR, mean rSO_2_ did not differ between groups; however, higher rSO_2_ during CPR was associated with ROSC and showed moderate discrimination for predicting ROSC (AUC 0.74).	rSO_2_ placement time was recorded and staff were trained; the rSO_2_ device was positioned out of the CPR performers’ line of sight, and in the MCCD group, manual compressions continued until the device was installed (15–20 s).	Neurological outcomes could not be assessed because no patients survived to hospital discharge. Cases achieving ROSC after short-duration CPR without rSO_2_ measurement or MCCD application were not included.
Storm 2016 [34]	To determine whether cerebral oxygen saturation measured during CPR has prognostic value.	ROSC; Neurological outcome	Very low initial cerebral oxygen saturation during CPR was nonetheless compatible with ROSC and a desirable neurological outcome, indicating that low starting values should not be treated as an absolute marker of poor prognosis.	An additional paramedic conducted trial-related monitoring to prevent interference, and sensors were placed during CPR or within two minutes after ROSC.	The rSO_2_ trajectory during resuscitation up to ROSC was not recorded. The NIRS signal was not exclusively cerebral, and extracerebral contamination could have confounded measurements.
Tsukuda 2019 [40]	To examine whether TOI is associated with ROSC and whether it could inform decisions to stop CPR or escalate to ECPR.	ROSC; CPR guidance/decision support	Initial TOI values were higher in ROSC than in non-ROSC patients and incorporating initial TOI improved discrimination between these outcomes. The authors framed TOI as potentially informative for prognostication and for decision-making around CPR continuation, termination, or escalation to ECPR.	Probes were applied within 30 s by the physician team leader without interrupting CPR.	This was a single-centre study with a small sample size, and the validity of the TOI cut-off values was not evaluated. Because NIRS devices use non-uniform proprietary algorithms, results may not be comparable across devices.
Asim 2014 [42]	To monitor cerebral oxygenation during CPR in OHCA patients using near-infrared spectrophotometry.	ROSC; Survival	Rises in cerebral saturation during CPR correlated with ROSC, supporting the premise that intra-arrest cerebral oximetry may provide a prognostic signal for ROSC and survival.	NIRS was not applied before admission due to the risk of disconnection; a nurse monitored and recorded saturation values.	The authors indicated that multicenter studies with larger patient numbers are needed. Neuroprotective agents and hypothermia were not used.
Singer 2015 [13]	To evaluate whether rSO_2_ measured during CPR is associated with ROSC and survival among cardiac arrest patients treated in the emergency department.	ROSC	Higher mean rSO_2_ during CPR was associated with a greater likelihood of ROSC, with ROSC rarely observed when rSO_2_ remained below 30% throughout resuscitation.	This approach was feasible, did not interfere with or interrupt care, and probe placement was comparable to applying a pulse oximetry pad.	Only patients presenting without ROSC were included, and analyses were limited to ED-arrival measurements rather than true arrest or CPR onset. The sample size was small with only one survivor; the device measured only the frontal cortex, and the study was single-centre.
Kalkan 2015 [25]	To compare initial versus end-of-resuscitation abdominal and cerebral saturation values in OHCA and assess whether increases in these measures correlate with ROSC.	ROSC	Changes in abdominal rSO_2_ over the course of CPR also carried prognostic information: a greater increase from start to end of resuscitation was significantly correlated with ROSC, and abdominal and cerebral saturation elevation values were themselves correlated.	NIRS was not applied before admission because of possible disconnection; values were visible to the team but not used for decision-making, and one nurse was assigned to monitor and record them.	The primary limitation was the small cohort. The duration of cardiac arrest before resuscitation was not determined, and its effect on hospital discharge remains unknown.
Meex 2013 [7]	To assess the practical feasibility of implementing NIRS monitoring during CPR.	Feasibility; Chest compression quality	Using FORE-SIGHT and EQUANOX systems, cerebral oxygen saturation (SctO_2_) monitoring was feasible during CPR after both IHCA and OHCA, and SctO_2_ dynamics appeared to vary with chest compression quality, suggesting sensitivity to resuscitation performance.	FORE-SIGHT required a third person to carry the device, whereas EQUANOX did not. To minimize delay, only one EQUANOX sensor was applied on the right forehead. Time to the first value was reported as ±10 s for EQUANOX and ±32 s for FORE-SIGHT. Signals remained stable for the first minute except in the two excluded patients. Protective adhesive tape was used to reduce interference from external light.	The study was a small pilot feasibility cohort, and not all patient or CPR characteristics were available. Two NIRS technologies with different proprietary algorithms were used; extremely low EQUANOX values (0%) were observed, and the authors discussed possible technical artifact interference.
Prosen 2018 [36]	To describe temporal changes in cerebral oximetry during OHCA resuscitation, with particular focus on the period surrounding ROSC.	ROSC	Initial rSO_2_ at CPR initiation was often extremely low (below 15%) but rose with ongoing resuscitation and was higher among those who achieved ROSC. A rapid, sustained rise was observed minutes before ROSC with normalization after ROSC, and no ROSC cases exhibited persistently low rSO_2_ punctuated only by transient spikes.	The median interval from arrival on scene to initiation of NIRS monitoring was 6 min (range 1–38 min); barriers included competing ALS priorities with limited personnel, and technical constraints such as restricted space, lack of a power source, and limited battery duration.	Convenience sampling and delayed sensor placement meant that not all eligible OHCA cases were captured, and not all patients had measurements throughout CPR. The INVOS device displays rSO_2_ only above 15% (values below are treated as 0), limiting analyses at very low levels.
Genbrugge 2018 [39]	To test whether increases in rSO_2_ during advanced life support in OHCA are associated with achieving ROSC.	ROSC	During prehospital ALS, higher intra-arrest rSO_2_ values and an absolute rSO_2_ increase of at least 15% were associated with ROSC, indicating that both absolute level and directional change conveyed prognostic value.	To reduce delay, a single sensor was used on the right forehead without skin preparation; the study was unblinded due to bedside confirmation of signal quality.	Not all eligible OHCA patients were enrolled. rSO_2_ and EtCO_2_ were measured on separate monitors, and clinicians were not blinded to rSO_2_, which could have influenced decisions to discontinue resuscitation.
Al-Subu 2020 [23]	To determine whether combined changes in two-site rSO_2_ and EtCO2 can evaluate resuscitation effectiveness and identify ROSC in a pediatric swine ventricular fibrillation model.	ROSC	Two-site rSO_2_ and EtCO_2_ tracked changes in cardiac output during CPR, and sudden increases in these signals identified ROSC without requiring interruption of resuscitation.	rSO_2_ and EtCO2 were measured during CPR and after ROSC using adhesive, noninvasive NIRS probes.	The open-chest model may have altered venous return and cardiac output; animals had relatively healthy lungs and received analgesic drugs, which may have confounded EtCO_2_ and related physiology.
Duvekot 2015 [58]	To identify OHCA patients at greatest risk of hyperfibrinolysis, with a specific focus on cerebral oxygenation measurements.	Hyperfibrinolysis risk stratification	Hyperfibrinolysis occurred more frequently when the initial cerebral TOI during resuscitation was 50% or lower, and this pattern was linked to higher t-PA levels.	NIRS monitoring was initiated immediately on ED arrival while CPR continued.	The study was single-center, observational, with a relatively small sample size (n = 46).
Frisch 2012 [29]	To assess continuous NIRS-derived StO_2_ monitoring during and after CPR and compare StO_2_ trends with ETCO2 for detecting ROSC or rearrest.	ROSC	StO_2_ fell prior to re-arrest or loss of pulses and rose rapidly with ROSC, supporting its potential utility for identifying ROSC during CPR and potentially reducing pauses for pulse checks; relative to EtCO_2_, StO_2_ also exhibited less variance.	The monitor was applied to patients who had already experienced ROSC those being transported to the hospital, and those expected to re-main in the resuscitation effort for a sufficient duration. physicians had no prior training and were instructed not to base decisions on the values. Time-to-placement, dislodgement, and interference were not reported.	This case series was limited by a small sample (n = 5) and by imprecise timing of pulse loss and return in the absence of an arterial line, with CPR start and stop used as surrogates for pulselessness. The StO_2_ monitor also produced more data points than the ETCO_2_ monitor, which may have influenced the apparent smoothness of the respective curves.
Deakin 2016 [51]	To characterize cerebral oximetry changes during in-hospital CPR and assess whether epinephrine administration improves cerebral tissue oxygenation.	Physiologic comparator; Epinephrine response	During CPR for IHCA, administration of epinephrine was associated with only a small mean increase in cerebral rSO_2_ over the subsequent five minutes and did not produce a meaningful change in the rSO_2_ slope when comparing the periods before and after dosing. Consistent with this pattern, the authors interpreted the early post-1 mg IV epinephrine interval as showing no clinically significant alteration in cerebral tissue oxygenation.	Median time to oximeter sensor placement was 5 (3, 7) minutes, and the duration of oximetry monitoring during cardiac arrest was 15.5 (8.3, 22.8) minutes; staff training and certification for data collection and REDCap entry were described.	CPR quality was not directly monitored, and the authors assumed it was similar before and after epinephrine administration. The five-minute “epinephrine-free” baseline may still have reflected circulating epinephrine, given its short half-life.
Putowski 2025 [27]	To compare rSO_2_ with ETCO2 during CPR and examine how each measure relates to ROSC and neurological outcomes.	ROSC; Survivals	rSO_2_ and EtCO_2_ measured during CPR were predictive of ROSC and survival, with rSO_2_ demonstrating greater predictive value than EtCO_2_ in the reported analyses.	Patients were excluded due to a lack of sensor connection (n = 31), and post-application exclusions included late sensor placement (n = 8), CPR discontinuation for palliative care (n = 4), electrode damage (n = 3), and device malfunction (n = 2); sensor calibration time was 10 s.	Proper CPR delivery was assumed, and brief, unrecorded interruptions may have occurred during ALS procedures. Because multiple confounders influenced rSO_2_, the study did not account for peri-resuscitation medications and did not examine rSO_2_ in relation to CPR decision-making, including termination or ECPR.
Sanz-Pescador 2024 [30]	To develop an approach for estimating chest compression rate in OHCA using features derived from the cerebral oximetry signal.	Chest compression rate estimation	A wavelet-based analysis of cerebral oximetry-derived ΔcHb oscillations during CPR can be used to estimate chest compression rate with low median absolute error and narrow 90% limits of agreement relative to thoracic impedance. The authors suggested that this approach could be implemented within existing cerebral oximetry monitors to provide real-time feedback on compression rate.	The method leverages existing high-temporal-resolution cerebral oximetry signals and can be implemented via software-only modifications to oximetry equipment without hardware changes.	Although overall accuracy was good, substantial errors could occur within individual 10 s windows when the spectral peak aligned with a harmonic, particularly near the 100–120 cpm guideline range or at high chest compression rates; the RA/Ra-based correction could fail or introduce miscorrections. Evaluation was limited to a single EMS dataset of 30 OHCA patients and focused on chest compression rate estimation, without clinical outcome validation or comparison to other feedback devices.
Pourzand 2024 [26]	To determine whether the combined strategy of head and thorax elevation, active compression–decompression CPR, and an impedance threshold device (AHUP-CPR) should begin early as BLS versus later as ALS in a severe porcine cardiac arrest model.	Mechanistic findings; Survival; Neurological outcome	In this severe porcine model of prolonged cardiac arrest, early initiation of AHUP-CPR yielded higher rSO_2_ and EtCO_2_ during CPR and in the early post-ROSC period, accompanied by improved hemodynamics, greater responsiveness to epinephrine, higher 24 h survival, and better neurological outcomes when compared with delayed transition to AHUP-CPR following an initial period of conventional CPR.	Not reported	The animals were young and otherwise healthy, differing from typical OHCA populations, and the first defibrillation shock was intentionally delayed, creating a highly severe model that may underestimate the potential for favourable neurologic outcomes with earlier treatment. Neurologic assessment was restricted to short-term (24 h) outcomes, with no evaluation of longer-term recovery, limiting inference about chronic outcomes.
Raymond 2024 [31]	To examine whether pediatric crSO_2_ measured by NIRS during CPR is associated with ROSC and survival to hospital discharge.	ROSC; Survival; Neurological outcome	In pediatric IHCA, higher intra-arrest cerebral oxygenation during CPR, along with spending more time above prespecified crSO_2_ thresholds (≥20–50%), was associated with higher rates of ROSC, survival to hospital discharge, and favourable neurological outcome. Notably, all patients who achieved ROSC, survival to discharge, or favourable neurological outcome maintained crSO_2_ above 30% throughout the resuscitation event.	NIRS monitoring followed routine clinical practice: at two sites, probes were placed on all patients at admission, whereas at one site probes were applied at the time of cardiac arrest; data were acquired using existing devices and BedMasterEX. The article describes cerebral NIRS as a practical noninvasive monitoring approach but does not report time to probe placement or quantify dislodgement or interference during CPR.	Only three centers contributed cardiac arrest events despite 56 hospitals in the collaborative, which may limit generalizability and reflect sites with a particular emphasis on CPR quality. Two NIRS devices (INVOS and Equanox) were used, and device-specific calibration and threshold differences may influence crSO_2_ values and limit the applicability of a single universal target.
Koyama 2023 [50]	To compare ScO2 patterns during ventricular fibrillation versus asphyxial cardiac arrest in porcine models.	Physiologic comparator; Etiology comparison	This study indicated that the physiological response captured by cerebral oximetry differed by arrest etiology: TOI rose substantially faster during CPR in a cardiogenic VF arrest model than in an asphyxial arrest model (16.6 vs. 1.1%/min), and TOI values from one to six minutes after CPR initiation were higher in VF arrest, paralleling higher rates of movement recovery following ROSC.	NIRS probes were placed bilaterally over each cerebral hemisphere; post-mortem dissection confirmed a scalp-to-brain distance of no more than 1.5 cm and indicated that the device penetration depth (3 cm) was sufficient for cerebral measurements.	The observation window was limited to 60 min after ROSC, and limb movement within one hour was used as a neurologic surrogate, which the authors noted was insufficient for a comprehensive neurologic assessment. The small sample size and protocol timing choices, including CPR initiation four minutes after cardiac arrest, further limit the generalizability of TOI–outcome relationships.
Jang 2023 [37]	To evaluate whether rSO_2_ in the first 5 and 10 min of CPR, relative to initial rSO_2_ and mean rSO_2_ across the full resuscitation, can help predict resuscitation futility in OHCA.	ROSC	In adult OHCA, the prognostic signal within cerebral rSO_2_ appeared to depend on temporal aggregation rather than single early snapshots. Highest and mean rSO_2_ values across the first five and ten minutes, as well as across the full resuscitation, showed moderate predictive performance and high specificity for non-ROSC, whereas initial single values were poor discriminators; persistent overall rSO_2_ ≤ 18% was uniformly associated with failure to achieve ROSC.	NIRS probes were applied to the forehead within one minute of ED arrival while CPR continued; rSO_2_ was recorded continuously until CPR termination or sustained ROSC, and values were blinded to clinicians. When the two probe readings differed, the lower value was used for prognostic analyses (except for the initial value), and no interruptions, dislodgements, or interference were described.	This was a small, single-center observational study within a specific EMS and hospital context, limiting generalizability and constraining multivariable adjustment. Only ED rSO_2_ during CPR was assessed, while prehospital rSO_2_ and long-term neurologic outcomes were not evaluated; accordingly, rSO_2_ thresholds should be considered within a broader multimodal framework rather than in isolation.
Košir 2023 [24]	To assess whether skeletal muscle rSO_2_ can be feasibly monitored during resuscitation and whether meaningful changes can be detected.	ROSC; Feasibility	Skeletal muscle oximetry during OHCA resuscitation was feasible, and both baseline and maximal skeletal muscle rSO_2_ values were higher among patients who achieved ROSC than among those who did not. The authors proposed that peripheral rSO_2_ may add information relevant to arrest duration and resuscitation efficiency.	NIRS probes were applied as soon as possible after ALS initiation, secured with additional tape on the right forehead and right thenar; monitoring was unblinded, but teams were instructed not to use rSO_2_ values for decision-making. The flowchart indicates feasibility, with 20 of 30 cases suitable for analysis after addressing signal and timing issues.	The study was limited by a small sample size and a single-center design. The small number of ROSC cases precluded analysis of temporal trends and the prognostic value of skeletal muscle rSO_2_ for favourable neurologic outcomes, and the study was not designed to assess rSO_2_ as a guide for post-resuscitation therapy.
Koyama 2013 [5]	To evaluate NIRS as a tool for assessing chest-compression quality in cardiac arrest and to determine its value for outcome prediction.	ROSC; Chest compression quality	During manual CPR in adult cardiac arrest patients, NIRS-derived ΔcHb waveforms tracked chest compressions in real time, and higher cerebral TOI both at emergency department admission and during ongoing CPR (using thresholds of TOI_adm ≥ 40% and TOI_CPR ≥ 50%) was significantly associated with ROSC.	NIRS provided synchronous ΔcHb waveforms and enabled real-time assessment during CPR.	The small cohort and absence of HbO_2_ and HHb measurements limited the evaluation of NIRS-derived metrics. All patients ultimately died despite some achieving ROSC, which the authors identified as a major limitation that may reflect procedural issues.
Parnia 2012 [10]	To assess the feasibility of deploying a commercially available cerebral oximeter during in-hospital cardiac arrest and to test whether cerebral oximetry predicts ROSC.	ROSC; Feasibility	Feasibility findings during IHCA supported intra-arrest cerebral oximetry acquisition, with higher rSO_2_ during CPR, considering both the overall resuscitation period and the final five minutes, associated with ROSC; the authors consequently suggested a potential role for cerebral oximetry in predicting ROSC.	Use was reported not to interfere with care, with placement time described as approximately 15 (±10) seconds, and CPR was not stopped during the process.	The authors noted that larger studies are needed, as findings were based on a small sample, and other CPR-quality parameters were not consistently available.
Bein 2006 [47]	To compare NIRS-based cerebral oxygenation with local brain tissue oxygen partial pressure during porcine CPR, and to determine whether measurements differ when optodes are placed on intact skin versus directly on the skull.	Physiologic comparator; Optode-placement effect	In the porcine VF/CPR/ROSC experiment, NIRS measurements were sensitive to optode placement, with signals acquired over the skull differing from those recorded over the skin. Skull-based NIRS correlated with ptiO_2_, and skin-versus-skull readings displayed transient dissociation following vasopressin administration during CPR, highlighting anatomical and pharmacologic influences on the measured oximetry signal.	Not reported	The study did not include a control condition without vasopressin, leaving the effect of AVP speculative. Interpretation of TOI was also constrained by the absence of a defined biological “zero,” and TOI values below 50 were noted to require cautious interpretation.
Bouček 2018 [9]	To examine whether cerebral and peripheral rSO_2_ measurements are associated with microcirculatory disturbances during cardiac arrest and CPR.	Physiologic comparator; CPR-quality indicator	rSO_2_ demonstrated greater responsiveness to the physiological effects of CPR than peripheral measurements. During mechanical CPR, brain rSO_2_ typically increased in a manner consistent with improved perfusion, whereas peripheral rSO_2_ did not show comparable changes, leading the authors to propose cerebral rSO_2_ as a potential indicator of CPR quality.	Application was rapid, use was simple and non-invasive, and the device was described as “easy-to-use.”	Baseline assessments showed variability in baseline values across individual animals. The study also compared intermittent, albeit frequent, rSO_2_ measurements with real-time microcirculatory and hemodynamic measures.
Putzer 2016 [44]	To characterize CPP, PbtO_2_, ScvO_2_, and rSO_2_ during CPR in a hypothermic porcine cardiac arrest model and quantify correlations between rSO_2_ and CPP, PbtO_2_, and ScvO_2_.	Physiologic comparator; Perfusion-pressure/oxygenation correlation	Physiologic coupling between perfusion pressure and oxygenation metrics appeared to depend on pharmacologic state. Prior to adrenaline administration, CPP, PbtO_2_, ScvO_2_, and rSO_2_ increased in parallel during chest compressions, suggesting broadly concordant changes in systemic and cerebral oxygen delivery. Following adrenaline, CPP and PbtO_2_ increased further while ScvO_2_ fell and rSO_2_ remained unchanged, indicating a divergence between global venous oxygenation, cerebral saturation, and perfusion pressure after vasopressor exposure.	Not reported	Absolute rSO_2_ values were not comparable across pigs, so analyses were performed relative to baseline. Correlations between rSO_2_ and CPP or ScvO_2_ were observed only when CPP and ScvO_2_ changed in parallel.
Lennmyr 2010 [28]	To investigate how cardiac arrest influences cerebral perfusion and oxidative stress under hyperglycemic conditions compared with normoglycemic conditions.	Physiologic comparator; Hyperglycemia effect	Post-resuscitation cerebral oxygenation was shaped by metabolic context, with cerebral rSO_2_ higher in hyperglycaemic patients than in normoglycaemic patients after ROSC (*p* < 0.05), suggesting that post-ROSC saturation values may reflect differences in systemic physiology beyond perfusion alone.	Not reported	rSO_2_ was described as a “summation index” with “superficial absorbance adjusted,” and as commonly reflecting deeper tissue oxygenation. Subcutaneous sensor placement may have influenced which tissues contributed to the measured signal.
Abramo 2014 [45]	To report two POHCA cases monitored with cerebral oximetry and BVI in the emergency department during arrest and post-arrest care, and to discuss potential prognostic implications.	CPR guidance/decision support	Cerebral oximetry incorporating a blood volume index (BVI) was described as a useful adjunct for resuscitation monitoring, particularly when conventional capnography became unobtainable. In these circumstances, cerebral oximetry was used to support decisions about whether to continue resuscitative efforts when EtCO_2_ was no longer recoverable.	Not reported	This study indicated a need for future research and stated that further investigation is warranted.
Kim 2022 [20]	To investigate the effect of the head-up position implemented during CPR on cerebral blood flow (CBF)	Physiologic comparator; Head-up positioning effect	In the emergency department, adopting a head-up position in which only the head and neck were elevated, without raising the chest, was associated with higher NIRS-derived cerebral blood flow and increased maximum cerebral blood flow velocity compared with the supine position.	NIRS data collection was challenging due to alternations between head-up and supine positioning; missing measurement values were reported, and patches were sometimes partially detached, which was addressed using processing rules.	NIRS data collection was challenging due to alternating head-up and supine positions, leading to missing values. NIRS was characterized as an indirect measure, with no clear evidence that it reflects forward flow, and scalp or CSF contributions were noted as potential influences on the measurements.
Ito 2014 [41]	To examine whether rSO_2_ measured on hospital arrival is associated with 90-day neurologic outcomes among OHCA patients.	Neurological outcome	rSO_2_ measured immediately after hospital arrival was associated with a good neurological outcome at 90 days and was used as a predictor in the study, with an optimal threshold reported at rSO_2_ > 42% across enrolled patients.	Two probes were applied immediately after hospital arrival, with a target of within three minutes; investigators were not blinded because monitoring required real-time visual confirmation. Exclusions due to inability to monitor rSO_2_ included insufficient personnel, probe shortages, and technical problems.	Investigators could not be blinded to rSO_2_ values, and rSO_2_ might have influenced decisions to stop resuscitation. Monitoring was limited to a short period after hospital arrival, and the lack of a portable device prevented it before hospital arrival.
